# A Multimodal Humidity Adaptive Optical Neuron Based on a MoWS_2_/VO*_x_* Heterojunction for Vision and Respiratory Functions

**DOI:** 10.1002/adma.202417793

**Published:** 2025-04-29

**Authors:** Abdul Momin Syed, Dhananjay D. Kumbhar, Hanrui Li, Manoj Kumar Rajbhar, Dayanand Kumar, Pratibha Pal, Nimer Wehbe, Mohamed ben Hassine, Nazek El‐Atab

**Affiliations:** ^1^ Smart Advanced Memory Devices and Applications (SAMA) Laboratory Electrical and Computer Engineering Computer Electrical Mathematical Science and Engineering King Abdullah University of Science and Technology (KAUST) Thuwal 23955–6900 Saudi Arabia; ^2^ Core Labs KAUST Thuwal 23955‐6900 Saudi Arabia

**Keywords:** crossbar array, heterojunction, high on/off ratio, humidity, in‐memory computing, memristor, multimodal, vanadium oxide

## Abstract

Advancements in computing have progressed from near‐sensor to in‐sensor computing, culminating in the development of multimodal in‐memory computing, which enables faster, energy‐efficient data processing by performing computations directly within the memory devices. A bio‐inspired multimodal in‐memory computing system capable of performing real‐time low power processing of multisensory signals, lowering data conversion and transmission across several modules in conventional chips is introduced. A novel Cu/MoWS_2_/VO*_x_*/Pt based multimodal memristor is characterized by an ON/OFF ratio as high as 10^8^ with consistent and ultralow operating voltages of ±0.2 surpassing conventional single‐mode memory functions. Apart from observing electrical synaptic behavior, photonic depression and humidity mediated optical synaptic learning is also demonstrated. The heterojunction with MoWS_2_ also enables reconfigurable modulation in both memory and optical synaptic functionalities with changing humidity. This behavior provides tunable conductance modulation capabilities emulating synaptic transmission in biological neurons while showing potential in respiratory detection module for healthcare application. The humidity sensing capability is implemented to demonstrate vision clarity using a convolutional neural network (CNN), with different humidity levels applied as a data augmentation preprocessing method. This proposed multimodal functionality represents a novel platform for developing artificial sensory neurons, with significant implications for non‐contact human–computer interaction in intelligent systems.

## Graphical abstract

A multifunctional memristor is demonstrated for in‐memory sensing and computing, leveraging a MoWS₂/VO_x_ heterojunction to enable high ON/OFF ratio up to 10⁸ with ultralow operating voltages of ±0.2 V. This bio‐inspired multimodal design exhibits tunable synaptic behavior across electrical, optical, and humidity stimuli, enabling in situ modulation of conductance for low‐power, real‐time processing of multisensory signals. The reconfigurable humidity‐adaptive neuron and humidity‐mediated optical synaptic learning enable non‐contact respiratory sensing and vision clarity control, paving the way for energy‐efficient next‐generation human–machine interfaces.

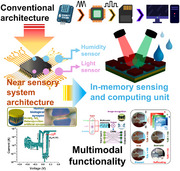

## 1 Introduction

Neuromorphic computing, inspired by biological brains, offers an efficient alternative to traditional CMOS‐based systems, addressing key computing bottlenecks and rising efficiency demands.^[^ ^1^ ^]^ Research and development of electronic biomimetic devices that mimic biological learning and memory are crucial for implementing neuromorphic computing.^[^ ^2^ ^]^ Artificial electrical synapses, vital for these systems, have been effectively replicated using non‐volatile memristors that function similarly to biological synapses.^[^ ^3^ ^]^ Non‐volatile memristors serve a crucial dual function in both data storage and computation, making them particularly effective for in‐memory computing applications.^[^ ^4^ ^]^ Furthermore, non‐volatile memristors can serve as sensory devices due to their capability for in‐memory sensing and computing and can be configured as artificial neurons through linear and nonlinear weight updates, thereby improving the stability of neuromorphic computing systems.^[^ ^5^ ^]^ Selecting suitable medium switching materials for innovative device designs is an effective approach to fabricating^[^ ^6^ ^]^functional memristors to multifunctional memristors.^[^ ^7^ ^]^

A multifunctional memristor significantly enhances the efficiency of both conventional and neuromorphic computing by mitigating the complexities inherent in hardware and software architectures.^[^ ^5e^ ^]^ This innovation facilitates simplified circuitry, improves energy efficiency, accelerates response times, and reduces processing costs, thereby optimizing overall system performance.^[^ ^8^ ^]^ Initially, memristors were primarily employed for memory applications due to their reduced dimensions, enhanced retention capabilities, and superior power efficiency.^[^ ^9^ ^]^ Subsequently, their proven potential has been explored in neuromorphic computing, as well as in data storage applications.^[^ ^10^ ^]^

Current research on multifunctional memristors remains limited, despite their promising potential in circuit design.^[^ ^11^ ^]^ A single memristor capable of multiple functions can simplify circuits, reduce energy consumption, and lower manufacturing costs. Such devices could integrate capabilities for data storage, sensing, and computation, offering significant advancements in innovative circuit architectures.^[^ ^11^, ^12^ ^]^ In advanced data storage applications, a unified system utilizing multifunctional memristors for both sensing and storage can improve performance and enable diverse data processing strategies.^[^ ^12^ ^]^ Additionally, these memristors can mimic the functions of artificial synapses and neurons in neuromorphic computing, potentially enhancing the efficiency of machine learning processes.^[^ ^12^ ^]^ However, research focusing on multifunctional memristors that combine capabilities such as electrical, optical, humidity sensing, and synaptic functions remains sparse.^[^ ^13^ ^]^ Additionally, in‐memory computing is very promising for sensing applications due to its non‐volatile behavior. Achieving a high on/off ratio, low power consumption, and a range of functionalities concurrently presents substantial challenges. To advance the development of versatile memristors for multifunctional circuits, further investigation into material selection, device architecture, fabrication techniques, and physical modeling is crucial.^[^ ^11^, ^14^ ^]^ Table S1, Supporting Information (SI) compares the key switching parameters for multimodal memristor devices. This research could unlock new applications and drive innovation in the field of electronics, paving the way for more efficient and capable devices in the future.^[^ ^11^, ^15^ ^]^ Based on our research, MoWS₂ demonstrates significant potential due to its tunable bandgap and superior charge transfer characteristics, yet it remains underexplored in the literature. The electrical properties of VO*_x_* based memristors have been widely studied^[^ ^16^ ^]^ Previously our group reported the optoelectronic synaptic behavior in Pt/VO/ITO‐based memristors.^[^ ^17^ ^]^ Building on this work, we have further advanced the development of a multimodal memristor by leveraging the synergistic effects of the VO*_x_* and MoWS₂ heterojunction. Building on the principles of in‐memory computing and in‐sensor computing, this research advanced in the field of in‐memory sensing, which converges the traditionally separate roles of analog sensors and digital memory into a unified, monolithic “MemSor” device.^[^ ^18^ ^]^

In this work, a multifunctional in‐memory sensing and computing device based on MoWS_2_/VO*_x_* heterojunction was developed. Our device showed multiple functions based on three different stimuli viz. electrical, optical, and humidity demonstrating its multimodal capability.  The device demonstrated excellent RS characteristics with an ultrawide memory window of 10^8^ at ultra‐low operating voltage of ±0.2 V. Apart from electrical potentiation and depression behavior, the devices were optically programmed to reduce the HRS using 532 and 635 nm lasers. Further, the RS characteristics were reversibly modulated by increasing and decreasing the RH humidity. Based on the tunable V_TH,_ we developed a humidity adaptive neuron and further demonstrated its ability for respiratory monitoring, targeting critical diseases like detection of chronic obstructive pulmonary disease (COPD). Furthermore, reconfigurable humidity mediated optical synaptic learning behavior was realized using 465 nm laser at different laser power intensity. It was employed to demonstrate the simultaneous effect of humidity and light on the vision clarity. The humidity mediated light sensing capability was implemented using CNN on the Oxford‐IIIT Pet Dataset, with different humidity levels applied as a data augmentation preprocessing method.

## 2 Results and Discussion

**Figure 1:**
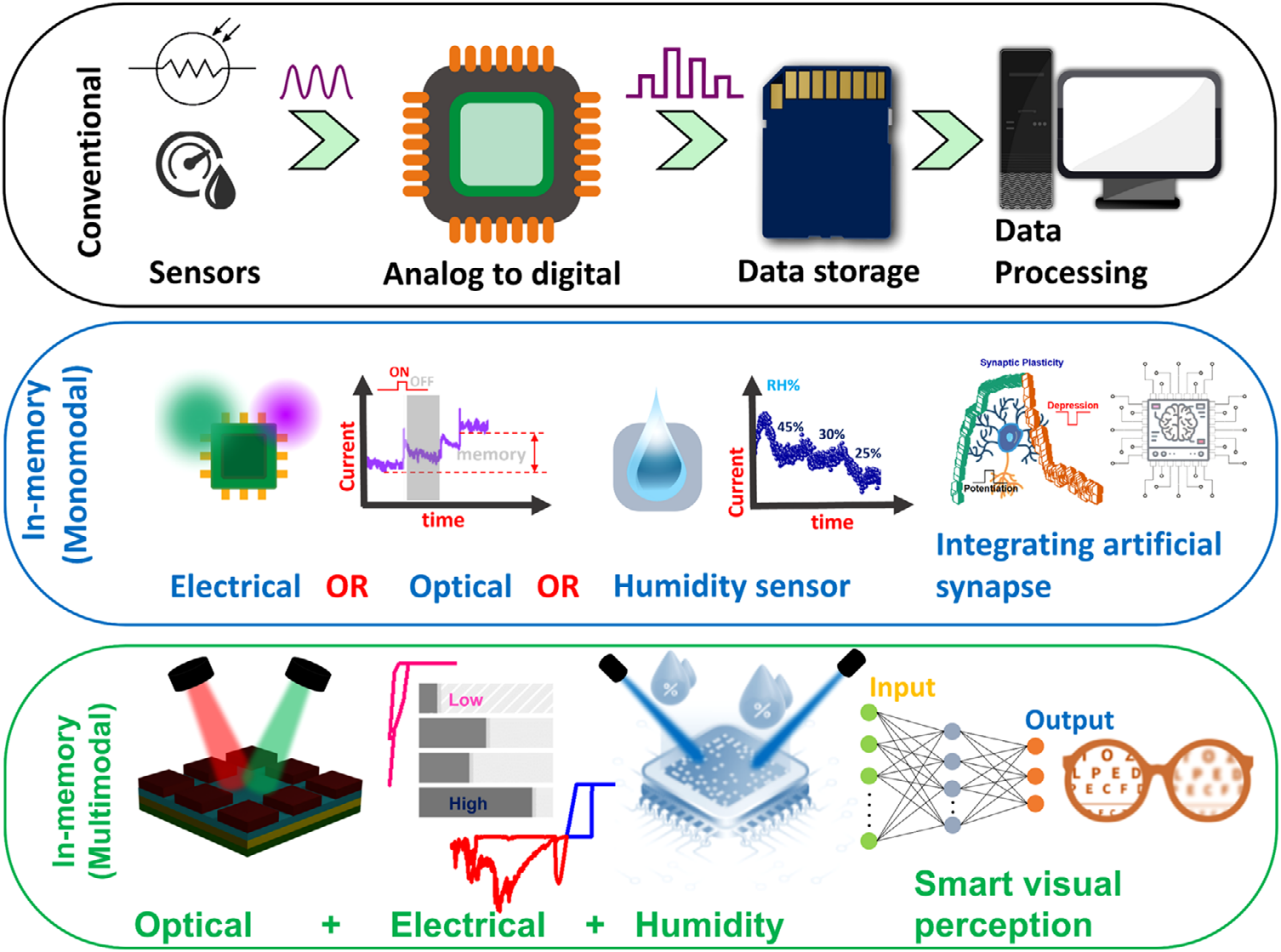
a) The analog signals from the conventional sensors are converted by the ADC chip into digital signals and then stored in memory. The data in the memory is loaded and transferred as the output signal by the processing units to the memory. b) Single stimuli sensitive memory computing (monomodal) integrates both sensing and storage functions; data can be sent directly to the processing unit, bypassing unnecessary intermediate steps. c) Multimodal devices possess respond simultaneously to different stimuli such as light, humidity, and show electrical synaptic behavior aside from its non‐volatile memory.

Most sensory systems rely on near sensor operations: for example, conventional systems comprise of humidity sensors for analog signal collection, analog‐to‐digital converters (ADC) for data transformation, memory units for storage, and processing units for calculations. This complex design necessitates advanced integration techniques, which increase latency and computational demands. A “More than Moore” approach, emphasizing functional diversification in sensing, storage, and data processing is desired in the present world. This shift encourages miniaturization by integrating non‐computing elements with digital technology for faster, energy‐efficient data handling. The progress started with single stimuli sensitive memory devices, and recent advancements in multifunctional sensory technology are revolutionizing data collection and processing, enabling the simultaneous capture of diverse information types and significantly enhancing system capabilities (**Figure** **1**). This work also aimed to develop an ultra‐low voltage operated memristor device that combines analog sensing and digital memory. This optoelectronic memory device can sense, store optical signals and respond to humidity at the same time.

**Figure 2:**
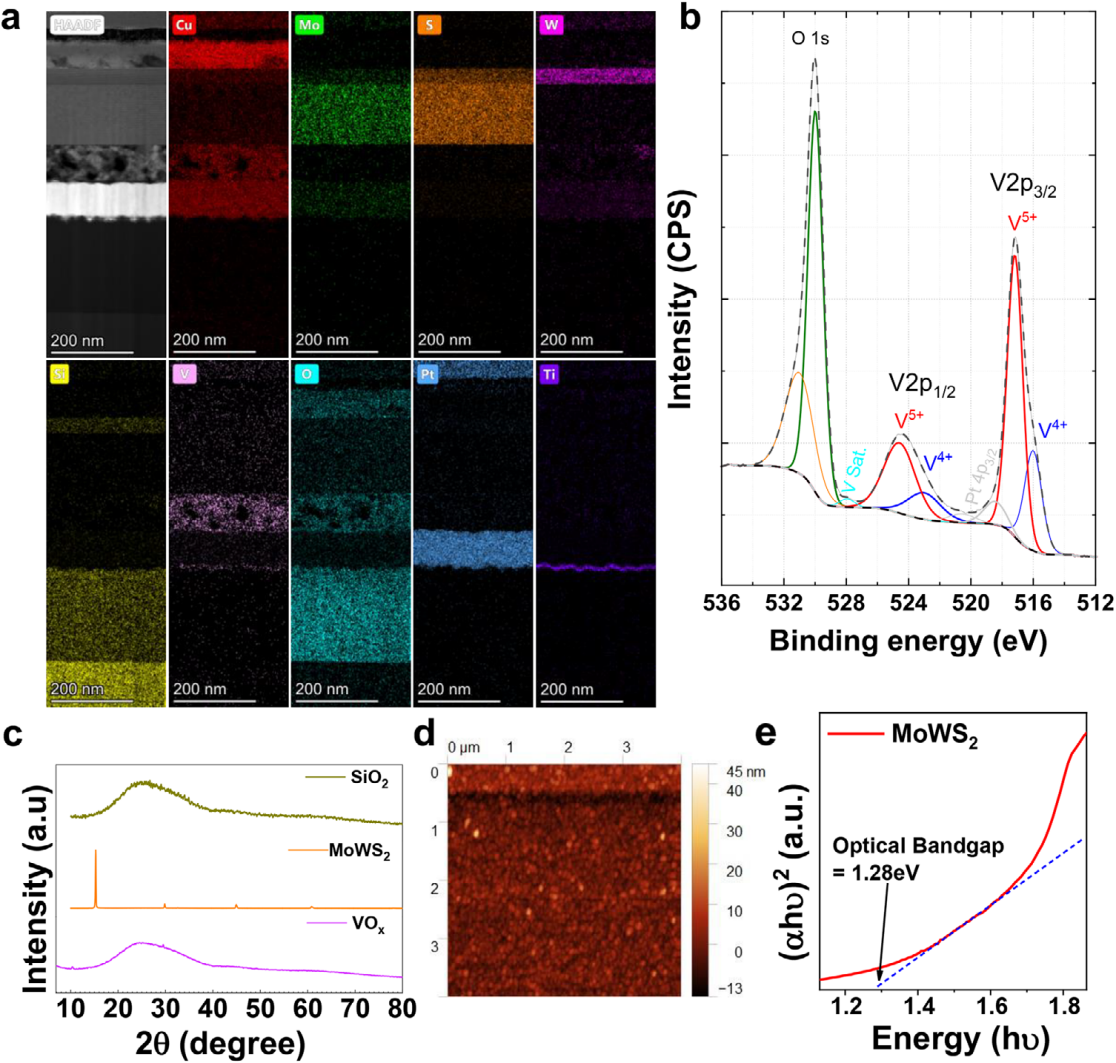
a) Cross sectional overview of the device; HRTEM and STEM – EDS mapping of the layers b) HRXPS spectrum of VO*_x_* thin film c) XRD spectrum of SiO_2_ (glass substrate used as a reference), MoWS_2_ and VO*_x_* thin films d) The surface morphology of the MoWS_2_/VO*_x_* film observed by AFM (e) optical bandgap of MoWS_2_.

The material characterization was performed to validate the composition of the MoWS_2_/VO*_x_* device. The cross‐sectional structure was observed under HRTEM and the elemental distribution within the layers was analyzed through STEM – EDS as shown in **Figure** **2**a. The EDS line profile validates the presence of copper (Cu), molybdenum (Mo), oxygen (O), Tungsten (W), vanadium, silicon (Si) sulphur (S), titanium (Ti) and platinum (Pt) in the device. The oxidation states in the sputtered VO_x_ were determined using high resolution X‐ray photoelectron spectroscopy (HRXPS). Figure S3, SI shows the survey spectrum of the VO*_x_* thin film deposited on the Si/SiO_2_/Ti/Pt stack. The high‐resolution spectrum of V 2p was acquired and curve‐fitted using casaxps software.^[^ ^19^ ^]^

Following the previous rules, we have obtained the peak fitting data of V 2p + O 1s illustrated in Figure 2b. As can be seen, the V 2p region is split into V2p_3/2_ and V2p_1/2_ where V2p_1/2_ appears noticeably shorter and broader than V2p_3/2_ due to a phenomenon known as Coster‐Kronig effect.^[^ ^20^ ^]^ The V 2p region could be deconvoluted into 4 major peaks located at binding energies of 516 eV for V2p_3/2_ (523 eV for V2p_1/2_) and 517.2 eV for V2p_3/2_ (524.6 eV for V2p_1/2_). These peaks can be assigned to V^4+^ and V^5+^, respectively which are the only detected forms of vanadium oxides meaning that oxidation states lower than V^4+^ in addition to V^0^ are absent.^[^ ^20^, ^21^ ^]^ The quantified data reveal that V^5+^ accounts for nearly 72% of the total vanadium oxides whereas V^4+^ represents the remaining 28%. It has been reported that vanadium oxides with oxidation state of 4 and lower will exhibit some multiple structures similar to the satellite we have fitted at 528 eV and that is due to V^4+^.^2,3^ In addition to these peaks related to vanadium oxides, we have discovered 2 more peaks detected at ≈518.5 and 520.6 eV. According to the literature, these values are high to be assigned to some forms of vanadium oxides. Knowing that our sample contains platinum, these two peaks overlapping with the vanadium region clearly denote Pt 4p_3/2_. Last, the oxygen O 1s peak can be fitted into 2 components detected at 530 eV and 531 eV. These peaks can be assigned respectively to vanadium oxide and some forms of carbon oxide contaminants. The calculated percentage of oxygen assigned to vanadium oxide is 67% and the remaining 33% is due to the oxygen introduced during the reactive sputtering. The XRD spectrum of the glass slide (SiO_2_), MoWS_2_, and VO*_x_* is shown in Figure 2c.

 The XRD spectrum of MoWS_2_ shows the (002) peak ≈14.5 degrees which corresponded to WS_2_ and exhibited a notably strong intensity.^[^ ^22^ ^]^ Additionally, (105) and (110) peaks with weak intensity corresponding to MoS_2_ were identified at 44.8 and 60.5 degrees, respectively.^[^ ^23^ ^]^ The EDX profile and the SIMS depth profile of MoWS_2_ flakes confirmed the presence of Mo, W, and S as shown in Figure S2b,c, SI. The XRD spectrum of VO*_x_* film revealed its amorphous nature which corroborated with the HRTEM results. The interfacial roughness between MoWS_2_ and VO*_x_* layers was characterized by atomic force microscopy (AFM) and the 2D AFM image is shown in Figure 2d, where the roughness root mean square (RMS) is 3.892 nm. Furthermore, a 3D topography and the line profile of roughness is shown in Figure S4, SI. The 3 arbitrary straight lines were drawn across the 2D AFM image of the heterostructure to study variations in the roughness across the different lines in the 4 × 4 um^2^ AFM scan. The consistent roughness observed in the line scans suggested good interfacial contact. The effect of relative humidity on the resistance‐switching characteristics of the device was examined using a multisensory setup housed with a microprobe station (Figure S4, SI). The optical bandgap of MoWS_2_ was observed to be 1.28 eV (Figure 2e) whereas that of VO*_x_* and the MoWS_2_/VO*_x_* heterojunction was found to be 2.63 and 2.56 eV, respectively (Figure S5, SI).

**Figure 3:**
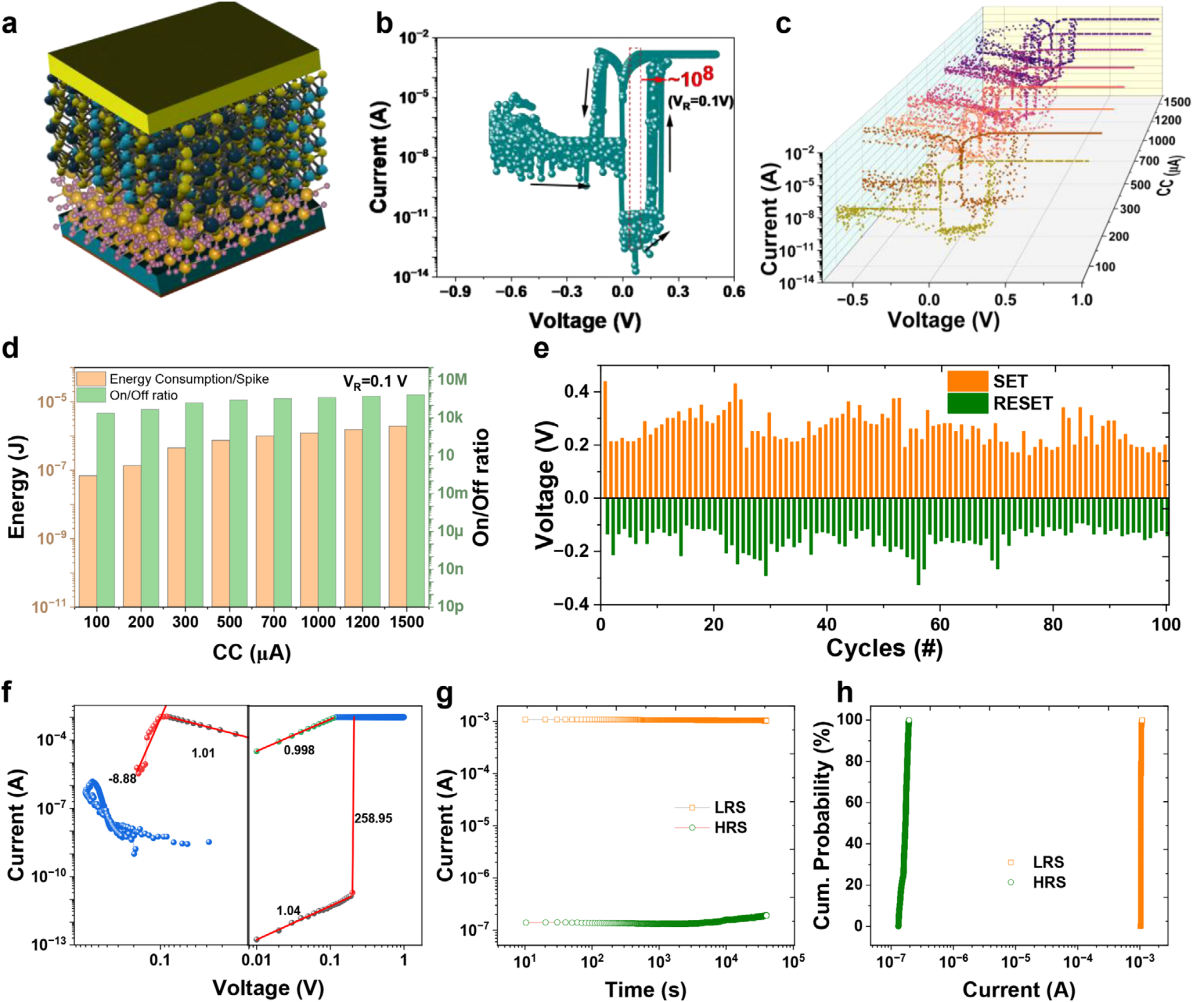
a) Schematic illustration of device structure Cu/MoWS_2_/VO_x_/Pt memristor device. b) 10 cycles of Current‐voltage characteristics in single logarithmic form of memristor device under compliance current (CC) of 1.5 mA. c) CC dependent multicyclic current voltage characteristics in single logarithmic form. d) Evaluation of CC based per read energy consumption and on/off ratio. e) Depiction of cyclic variation in the switching parameters of memristor devices as V_SET_, V_RESET_, I_SET_ and I_RESET_, respectively. f) Double logarithmic plot of current voltage characteristics to reveal the conduction mechanisms of resistive switching. g) Retention of I_on_ and I_off_ states measured till 40,000 s and h) cumulative probability of respective the resistance states.

The electrical performance of Cu/MoWS_2_/VO*_x_*/Pt‐based memristors, depicted in **Figure** **3**a, was evaluated through cyclic DC voltage sweeping. In this configuration, the copper top electrode was consistently biased, while the platinum bottom electrode remained grounded. To initiate the resistive switching behavior, an electroforming process was conducted by sweeping the voltage from 0 to 4 V, with a step increment of 0.01 V (Figure S6, SI). Following the electroforming step, the device was reset to the high resistance state (HRS) by applying a voltage of −0.7 V. Subsequently, the memristor was utilized for operational testing. The voltage sweep for device operation was applied within the range of −0.7 to 1 V, starting at 0 V, while a compliance current (CC) of 1.5 mA was enforced during the positive sweep to prevent device breakdown. As presented in Figure 3b, the current‐voltage (I‐V) characteristics of the MoWS_2_/VO*_x_*‐based memristor were measured across 10 consecutive cycles under consistent measurement parameters. The resulting I‐V curves demonstrate a butterfly‐shaped loop, characteristic of bipolar memristors. This behavior can be ascribed to the formation and rupture of conductive filaments, driven by the migration of oxygen vacancies, sulfur vacancies, and Cu ions in response to alternating positive and negative voltage biases.^[^ ^24^ ^]^

Notably, a sharp increase in current was observed as the applied voltage reached values between 0.16 and 0.21 V, indicating the SET process, whereby the memristor transitioned from the HRS to the low resistance state (LRS). The LRS was maintained throughout the return voltage sweep from 1 to 0 V and the subsequent application of a negative voltage. During the negative voltage sweep, when the applied voltage approached −0.7 V, the current exhibited a marked decrease, signaling the return of the device to the HRS and the completion of the RESET process. To modulate and optimize the resistive switching properties of memristor devices, variation in the compliance current (CC) is systematically investigated. CC optimization is critical for controlling the maximum current during switching events, which prevents excessive power dissipation, mitigates the risk of device damage, and ensures stable and reliable performance in memory applications. As shown in Figure 3c, the CC was varied between 100 µA and 1.5 mA (8 levels), with each level tested over 10 consecutive DC cycles to assess reliability and variability. The results demonstrate that across all CC levels, the devices exhibited consistent switching behavior with minimal variability, confirming reliable performance throughout the cycling tests. Notably, this memristor exhibited an exceptionally low off‐state current of ≈2 pA, coupled with a commendable ultrahigh on‐off ratio of 10^8^ (Figure 3b), a characteristic not observed in previously reported bare VO*_x_* – based memristor devices.^[^ ^17^ ^]^

CC directly affects both the energy consumption and the On/Off ratio in memristors. A higher CC allows more current to flow during the switching process, which can affect the growth of filament formation, leading to a higher On/Off ratio and increased energy consumption due to higher power dissipation. Conversely, a lower CC restricts the current, resulting in a lower current flowing, which decreases the On/Off ratio and reduces energy consumption as depicted in Figure 3d. Thus, optimizing CC is crucial for balancing energy efficiency and achieving the desired On/Off ratio, which is key for effective performance in memory and logic applications. Figure 3e illustrates the statistical analysis of the set and reset voltages and currents over 100 consecutive switching cycles. The cycle‐to‐cycle variability of the Cu/MoWS_2_/VO_x_/Pt memristors was assessed by measuring the switching parameters across these cycles and subsequently calculating the coefficient of variation (CV), which is expressed as the ratio of the standard deviation (σ) to the mean (μ). Complementary to this, Figure S9a, SI provides histograms depicting the distribution of set and reset voltages and currents, derived from aggregating the data of the 100 cycles. The mean (μ) and CV for the set voltage are recorded as 0.26 V and 0.059, respectively, while for the reset voltage, they are −0.15 V and 0.045, respectively. Figure 3e, Figures S7 and S8a, SI confirm the stability of the bipolar resistive switching (RS) behavior over multiple cycles, exhibiting relatively low variability in switching voltages compared to other memristive devices, both with and without high on/off ratios.^[^ ^25^ ^]^ Figure S8b, SI compares the ON/OFF ratio of various other devices against the MoWS_2_^/^VO*_x_* heterojunction device developed in this study. Device‐to‐device (D2D) variability represents a critical factor that warrants attention in the evaluation of memristor devices. To investigate this variability, 16 randomly selected devices were subjected to multi‐cyclic DC I‐V characterization, as illustrated in Figure S11, SI. The variability in the switching voltage across the devices is depicted in Figure S12, SI showing minimal variation in the switching voltage. Due to the solution‐processed nature of these devices, the study demonstrates an acceptable level of D2D variability in the switching cycles (HRS state). The variability has been attributed to the uncontrollable defect density and the stochastic behavior of filament formation on these defects.^[^ ^4^, ^26^ ^]^

To investigate the conduction mechanism in high on/off ratio Cu/MoWS_2_/VO_x_/Pt memristors, the double logarithmic I‐V characteristics, as depicted in Figure 3f, were analyzed using absolute voltage‐current values. By fitting specific regions of the curves with linear functions, it was determined that both the low resistance state (LRS) and high resistance state (HRS) exhibit similar conduction mechanisms. In the positive voltage region, both LRS and HRS display Ohmic conduction behavior, as indicated by a slope value of ≈1. However, in the transition region, a sharp and abrupt increase in the slope to ≈258 suggests conduction dominated by thermally generated carriers and space‐charge‐limited current (SCLC) at higher applied voltages. This observation further supports the formation of resistive switching (RS) filaments within the memristor. In contrast, under the negative voltage region, the device maintains its LRS until a lower threshold voltage is reached, at which point the conduction transitions to an Ohmic regime with a negative slope of ≈9, indicating filament rupture and the transition to the HRS. This HRS follows the Ohmic conduction mechanism. The cyclic and reliable observation of this phenomenon over consecutive switching cycles confirms the robust bipolar switching behavior of the Cu/MoWS_2_/VO_x_/Pt memristors. The proposed RS mechanism is schematically illustrated in Figure S13, SI.

The retention of resistance states of the devices, recorded at 0.1 V, is presented in Figure 3 g, while the corresponding cumulative probability distribution is shown in Figure 3h. These measurements provide insight into the stability and reliability of the Cu/MoWS_2_/VO*_x_*/Pt memristors over repeated switching cycles and stability of resistance states recorded over 40 000 s confirms the non‐volatile memory nature. This reliability is critical for practical applications, as it underscores the long‐term operational stability of the memristors.

**Figure 4:**
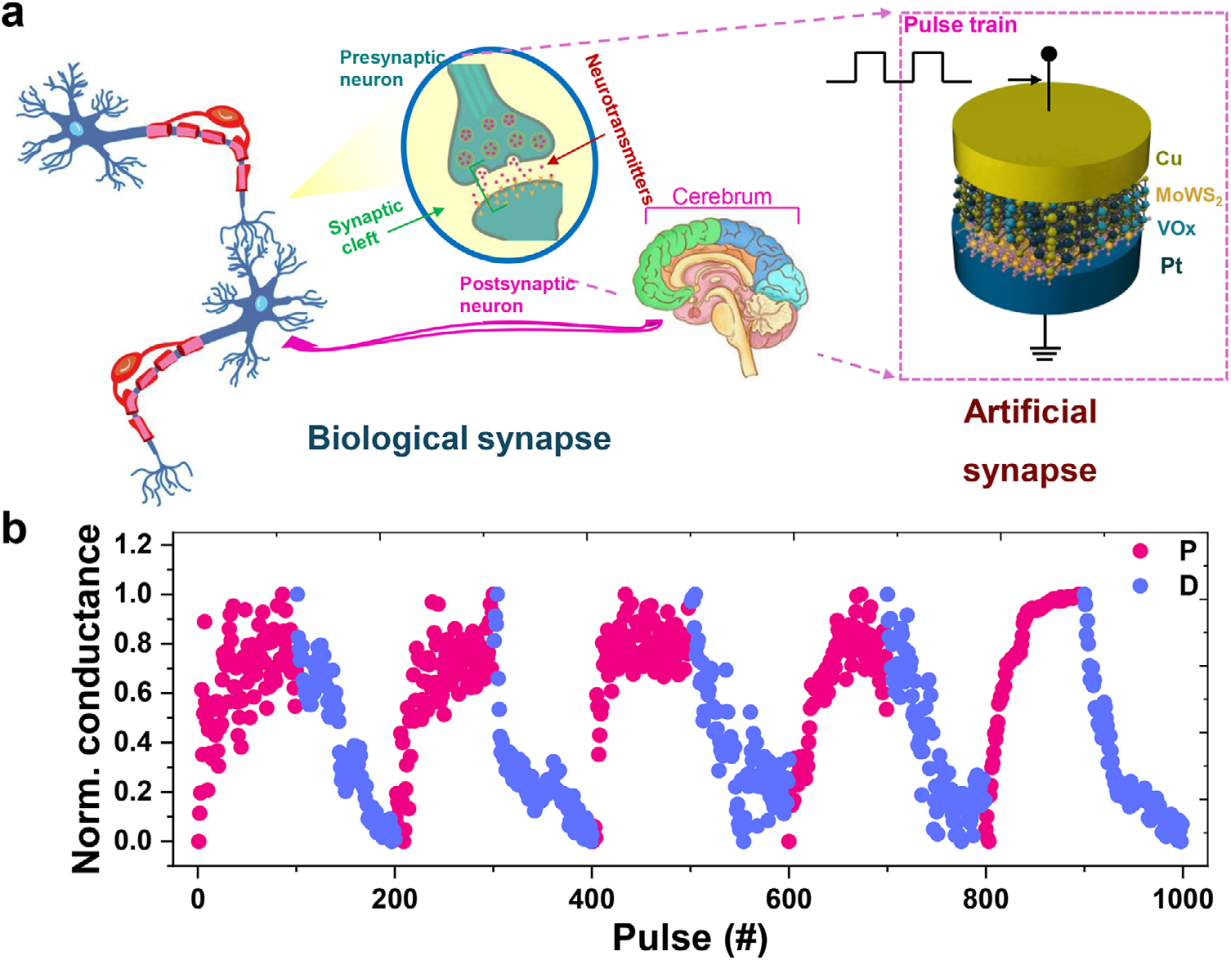
a) Schematic illustration of neurotransmission between biological neurons through synaptic cleft between pre and post neurons and proposed physical device‐based neurotransmission through the fabricated Cu/MoWS_2_/VO*_x_*/Pt based memristor device. b) Potentiation and depression characteristics of the memristor devices measured under repetitive pulse train of V_Write_ = 1 V, V_Erase_ = −1.2 V, and V_read_ = 0.1 V with psulse width = 200 ns and pulse interval of = 200 ns, respectively.

Biological neurons communicate by transmitting electrical signals through synapses, where the strength of these synaptic connections can be dynamically adjusted in response to learning and experience. Analogously, memristors exhibit similar behavior by modulating their resistance in response to an applied voltage, rendering them ideal candidates for replicating synaptic functions within artificial systems (**Figure** **4**a). Much like biological neurons, memristors possess the ability to store and process information simultaneously, which makes them highly promising for advanced computational tasks such as signal processing and image recognition. Among the various types of memristors, filamentary resistive switching (RS) is of particular interest, as it operates via redox reactions and ion migration in active metals to form or rupture conductive filaments (CFs), thus modulating the device's resistance. A key challenge in this domain is the development of memristors that offer both neuron‐like conductance updates and a high on/off ratio. Successfully addressing this challenge would significantly enhance the performance of neuromorphic computing systems, making it a critical objective in the field.

Long‐term potentiation (LTP) and long‐term depression (LTD) are artificial synaptic functions that can be effectively replicated using Cu/MoWS_2_/VO*_x_*/Pt memristors. Upon the application of 100 consecutive pulses, each with a duration of 100 ns, the postsynaptic current exhibits a progressive increase in response to positive pulses, whereas it decreases following negative pulses (Figure S14a, SI). This dynamic behavior leads to the emergence of asymmetric LTP and LTD behaviors depicted in Figure 4b, closely approximating the learning and forgetting processes characteristic of biological neurons. Moreover, the memristor demonstrates low energy consumption, with each spike requiring ≈5–9 pJ when exposed to 100 ns pulses (Figure S14b,c, SI), thereby achieving energy efficiency comparable to that of synaptic activity in neural systems. As illustrated in Figure S13c–e, SI, the nonlinearity coefficients for LTP and LTD behaviors range from 0.019 to 0.0465 for LTP and from −0.021 to −0.041 for LTD, respectively. The nonlinearity in LTP and LTD, as quantified by these coefficients, is required in effectively modeling synaptic plasticity.^[^ ^2^, ^27^ ^]^ Specifically, these coefficients regulate the scaling of synaptic strength modifications, thereby facilitating precise control over the dynamic interplay between potentiation and depression. This delicate balance is indispensable for replicating biological learning mechanisms and ensuring reliable memory stabilization in neuromorphic systems.

**Figure 5:**
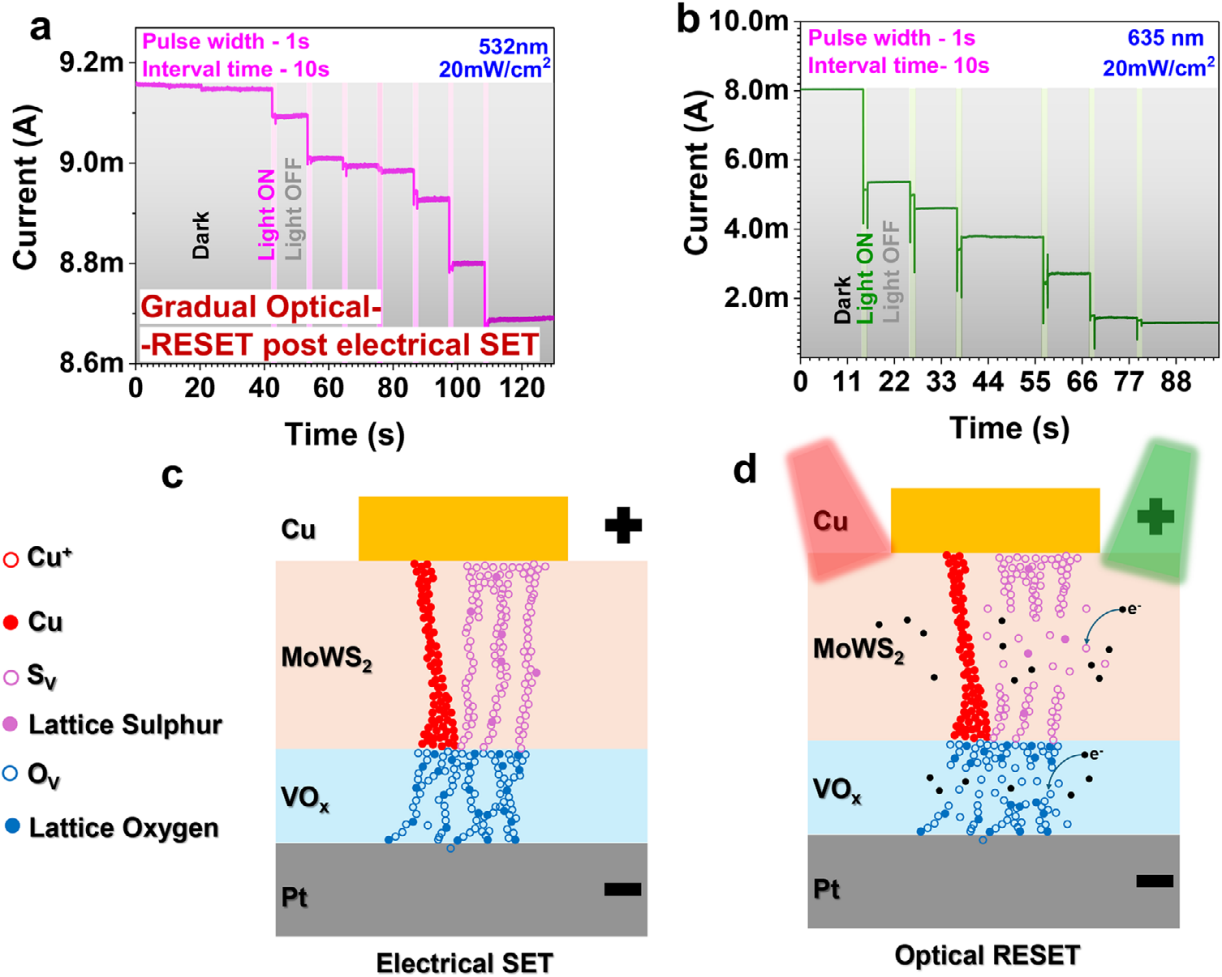
a) Current versus time curve for the device during light induced RESET followed by 532 nm, and b) 635 nm of wavelengths, respectively. Proposed schematic representation of c) electric set and d) optical reset mechanism of the devices.

Research has shown that electrically induced vacancies create energy states within the bandgap of resistive switching materials. Consequently, natural vacancies and adjacent interstitial ions fail to recombine during the electrical set process due to an energy barrier that hinders recombination. Although electrostatic forces favor rapid recombination of ion‐vacancy pairs, this energy barrier prevents it. Several researchers have reported that under illumination, a light source can supply sufficient energy for interstitial ions to recombine with electrically generated vacancies within the resistive switching layer. To comprehensively investigate the optical reset phenomenon, the device was initially electrically configured and sustained at a read voltage of +0.1 V to ensure its stable retention. The reset mechanism was corroborated by observing the transition in the device's resistance state from LRS to HRS upon optical stimulation. **Figure** **5**a,b demonstrate the effects of optical illumination, characterized by a pulse width of 1s and a pulse interval of 10 s, utilizing light sources with wavelengths of 532 and 635 nm, respectively. The experimental results unequivocally demonstrate that the optical stimulus alone induces the rupture of the conductive filaments. During the pulse intervals, in the absence of optical stimulation, the device maintains its resistance state. Both wavelengths (532 nm and 635 nm) were effective in filament rupture and inducing degradation of the LRS. Nevertheless, under identical measurement conditions, the 532 nm wavelength exhibited substantially lower degradation in the RS properties. Conversely, the 635 nm stimulus caused a significant degradation, with the current decreasing from 8 mA to ≈1 mA. The proposed mechanisms for the electrical SET process and optical reset effect are illustrated in Figure 5c,d. Upon illumination, the filament gets ruptured, causing the resistance to increase, and the current to decrease though it did not reach the HRS level of 100 nA. This suggests partial disruption of the filament, with Cu playing a dominant role in its formation and stability, while oxygen and sulfur vacancies contribute less. The illumination‐induced vacancy recombination causes partial filament disruption, but the Cu‐based filament remains intact, demonstrating the robustness of Cu in maintaining the conducting path. Additionally, the optically induced breaking of the filament likely involves the disruption of vacancy‐based filaments, a phenomenon also reported in previous studies.^[^ ^28^ ^]^ This observation suggests that the optical reset mechanism is primarily associated with modifications in the ionic species rather than alterations in the metallic filament, thereby influencing the observed degradation in RS characteristics.

The proposed optical reset mechanism presents numerous advantages, including the gradual resetting of resistance states and the potential for wavelength‐specific control, which may enhance scalability and reduce power consumption in resistive switching devices. Furthermore, this optically modulated tunability  is expected to facilitate faster switching speeds and improve reliability by minimizing thermal stress, thereby advancing the development of next‐generation memory applications.

**Figure 6:**
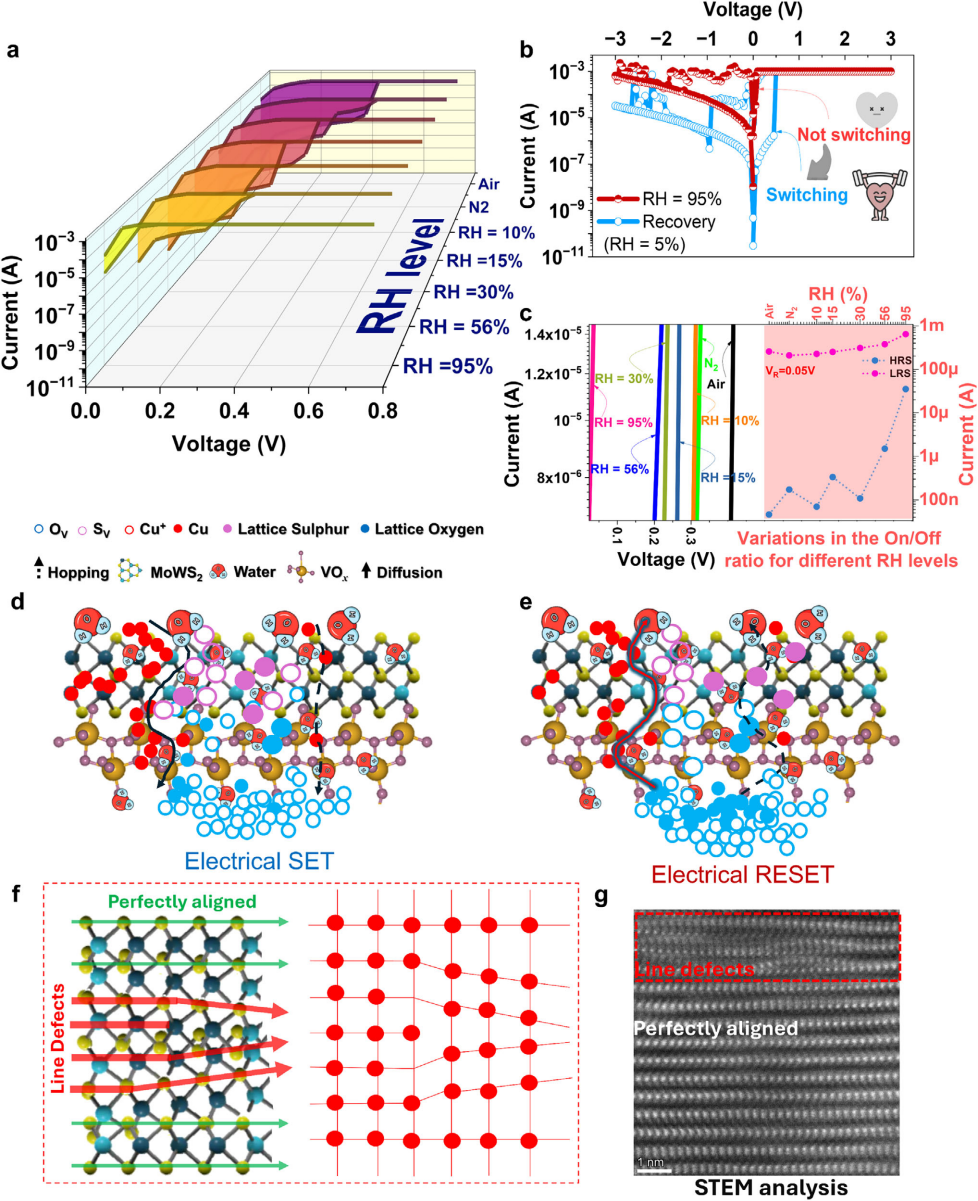
a) I–V curves measured at different RH levels b) I–V curves at RH = 95 and 5% demonstrating reversible switching c) Zoomed in graphs (from Figure 6a) showing the lowering of V_TH_ and effect on the On/Off ratio under the influence of various RH levels. Plausible RS mechanism under the influence of RH d) in SET and e) RESET mode () schematic illustration of the line stacks in MoWS_2_ lattice g) HRSTEM images of MoWS_2_ (scale = 1 nm) film showing line defects.

The memristor was next placed in a chamber with controlled N_2_ and H_2_O supply to analyze the effect of humidity on the switching behavior (Figure S15, SI) The humidity mediated resistive switching (RS) characteristics were investigated by varying the humidity from 10 – 95% (T = 25 °C). The RS curves at different RH levels are shown in **Figures** **6** and S16a, SI. The humidity sensing measurements revealed meaningful variations in the RS characteristics, particularly with reduction in the threshold voltage (V_TH_) during the SET program. As the device was exposed to complete N_2_ atmosphere, the V_TH_ lowered to 0.31 V from its initial value of 0 .38V. In line with the literature,^[^ ^29^ ^]^ we noted a decline in the conductivity of the VO*_x_*/MoWS_2_ memristor in a completely dry nitrogen atmosphere, which then increased in a 95% RH environment. As the humidity in the chamber gradually increased, the V_TH_ began to decrease, and the HRS curve also shifted upward. The device switched until the chamber became extremely humid (RH = 95%). Nonetheless, as the RH was further increased to 95%, the hysteresis curve narrowed significantly showing nearly conductive behavior as a result of extreme leakage current^[^ ^30^ ^]^ Figure 6c shows the shift in the V_TH_ which dropped from ≈0.41 V (in air) to ≈0.025 (at RH = 95%). In contrast, I_RESET_ exhibited less sensitivity to the humidity than I_SET_ and showed insignificant difference as the humidity was increased. This may be because the conductivity at negative voltage is influenced by the bottom electrode, which is less exposed to the humidity than the top electrode, resulting in reduced sensitivity.^[^ ^29^ ^]^ Only at RH = 95%, a higher I_RESET_ was observed, which also mirrors the drastic change in I_SET_. The reversibility of humidity sensing after losing the typical memristor loop in the SET region was also observed. At RH = 95%, the device remained in the LRS; yet interestingly, the memristor recovered successfully as the humidity was lowered to RH = 5% (Figure S16b, SI) and 20% (Figure S16c, SI). Multiple I‐V curves were swept at low, moderate, and high humidity. Figure S16d, SI shows the ten I‐V curves each swept at RH = 5, 56, and 95% with very little cycle to cycle variation. This also confirms the changes in RS characteristics observed with varying the humidity level were not merely stochastic (as C2C variability is otherwise commonly observed in memristors)^[^ ^31^ ^]^ but influenced by the humidity.

The increased conductivity of the memristor could be understood by Grotthuss conductivity mechanism as the proton tunnels between the water molecules through hydrogen bonding.^[^ ^32^ ^]^ We understood that the device conductivity was enhanced due to the surface mechanism where water molecules can fill carrier traps within the conductive filament.^[^ ^30^ ^]^ When the VO*_x_*/MoWS_2_ based memristor was exposed to humid environment by increasing the RH levels, the water interacts through the chemisorbed, and physisorbed layer(s). VO*_x_* is a transition metal oxide with a high affinity for oxygen, and defects in the VO*_x_* film as observed by HRTEM (Figure 2a), acted as active sites for molecular water adsorption. Interestingly, the water adsorption properties of VO*_x_* differ from those of V metal. Vanadium oxide has higher affinity toward molecular water adsorption and less toward hydroxide formation. A careful study by Dana et al.^[^ ^33^ ^]^ suggested that the work function of vanadium oxide film changed in dry conditions which was triggered by the electron transfer between the surface and the adsorbate. It could be due to the formation of a surface dipole layer, where the positive charge orientation is directed away from the substrate. This phenomenon showed consistency with the adsorption of H_2_O or OH⁻ molecules, wherein the O^2^⁻ binds to the surface, while the H^+^ extended outward, creating a dipole effect which facilitated electron transfer.^[^ ^33^ ^]^ Structural imperfections, dislocations/faults contributed to the formation of dangling bonds, as confirmed by HRTEM observations (resolution = 10 Å), which revealed stacking faults in the MoWS₂ structure. (Figure 6g). Highly crystalline 2D materials like WS_2_ are less sensitive to humidity due to lesser crystalline imperfections.^[^ ^34^ ^]^ Nonetheless, the dangling bonds at the edges of MoWS₂ in the lateral plane acted as active sites and the line defects (Figure 6e,f), increasing its sensitivity to humidity.

These localized electronic states allowed the charge hopping to the water molecules, facilitating charge transfer process. Water and molecular oxygen from the immediate surroundings attached to these edge defects lowering the barrier of cationic migration.^[^ ^35^ ^]^ But the electrostatic attraction between protons and V_Ö_ restricted the growth of the V_Ö_ based conductive filamfent. As a result, it led to a notable decrease in the ON/OFF ratio as the RH was increased.^[^ ^13^ ^]^ A report^[^ ^32^ ^]^ related the reversibility of WS_2_ based humidity sensor to the hydrophobic nature of the active layer. Another exhaustive study on the effect of relative humidity on the electrical characteristics^[^ ^31^ ^]^ explained that hydrophobic nature is not necessarily responsible for the reversibility. Regardless of the extent of the hydrophobicity, humidity will always accumulate at the tip‐electrode junction. The water meniscus present in the tip surroundings increases the effective area for the higher carrier injection. This interfacial accumulation of water increases the total contact area affecting the conductivity, DOS, and increases the ionic adsorption and the diffusion performance.^[^ ^36^ ^]^ At high humidity, higher leakage currents shrink the hysteresis in the I‐V curve. Reversibly when the humidity decreases significantly, the tip‐electrode junction becomes relatively drier and revives the RS behavior across the memristor. Figure S17, SIschematically represents the effect of increasing the RH level on the water meniscus present across the tip‐junction and the formation of multiple conductive filaments in the device. Mathematically, the currents measured by the tip under different RH levels could be expressed as *I*_RH%_  =  *A_c_* × *J* + *A_m_* × *J_m_*, where *A_m_*  = area with tip – moisture – Cu structure across which electrons flow, and *J_m_*  = average current density across *A_m_*. However, as correctly mentioned in recent report,^[^ ^31^ ^]^ the difficulty lies in calculating *A_m_* and *J_m_* because it does not merely depend on RH, but also on the thickness of the water meniscus on tip and sample surface which are affected by the wetting properties of the tip and sample.

As mentioned earlier, this humidification/dehumidification is responsible for the apparent increase/decrease in the currents (HRS), which is based on the explanation provided by reports.^[^ ^31^, ^33^ ^]^ Therefore, we assume that the reversibility of the memristor in regaining hysteresis and switching back at low RH after exhibiting nearly conductive behavior at high humidity likely stems from the catalytic effects of water in accelerating the conductivity, rather than from a water‐splitting reaction or hydrophobicity. Given that the operating voltage of the VO*_x_*/MoWS_2_ device is quite low (<0.25 V), this likely signaled the absence of any water‐splitting effect.^[^ ^29^ ^]^

**Figure 7:**
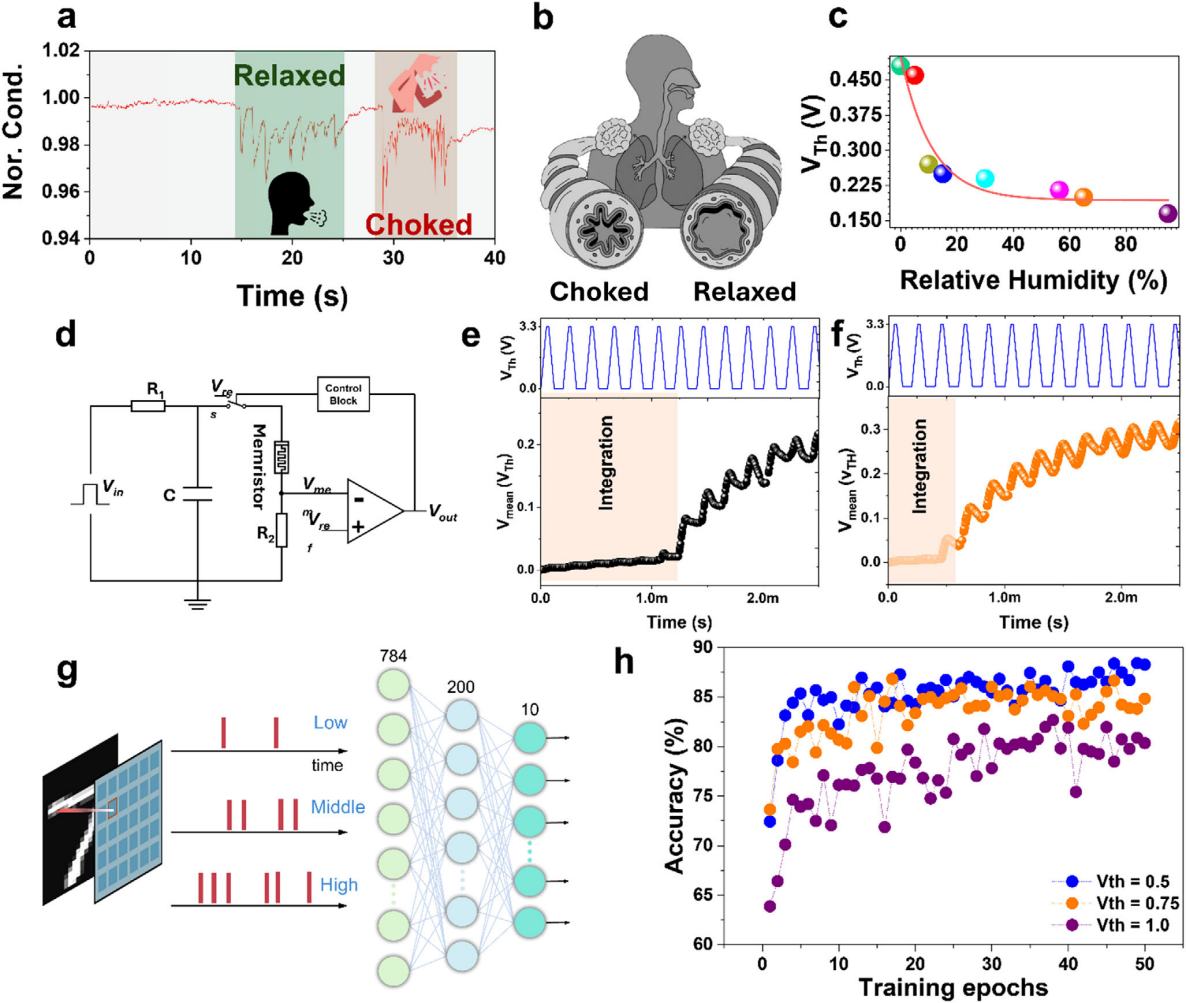
a) The distinguished current response based on the breathing patterns b) Relaxed (healthy) and choked (COPD) bronchi c) V_TH_ plotted against different RH values d) The circuit diagram designed for the humidity adaptive neuron The SPICE simulation results at the RH levels of e) 0.25 and f) 0.5 g) Spike encoding scheme h) Classification results for the spike neural network (SNN) with a 784‐100‐10 architecture.

The MoWS_2_/VO*_x_* memristor demonstrated the ability for respiratory monitoring, targeting critical diseases like COPD. During testing, the memristor recorded current responses to two breathing patterns —normal, and rapid. The current response is distinguished based on the breathing pattern (**Figure** **7**a) and exhibits a unique I–t profile. Crucially, the memristor differentiated the mimicked pattern of breathing for healthy individuals from COPD patients, who exhale drier gas due to airway obstruction and show shorter inspiratory and expiratory times (Figure 7b). Our device demonstrated threshold tuning ability according to the environmental humidity, as shown in Figure 7c. The fitting result shows the threshold value of MoWS_2_/VO*_x_* memristor decayed exponentially with the increase in humidity, suggesting its potential application as a humidity adaptive neuron. The threshold switching memristor enables the configuration of artificial neurons in bio‐mimic system with threshold switching (TS) and non‐volatile characteristics. The tunable threshold value according to humidity constructs the neuron with different encoding abilities in different environments. Figure 7d shows the circuit diagram of the humidity‐perception neuron proposed in this study. The VO*_x_*/MoWS_2_ memristor is connected in parallel with a capacitor and in series with a 2k Ω load resistor R_1_. Besides, a 500 Ω resistor R_2_ is utilized to protect the circuit and convert the current into a voltage output. Once the V_mem_ exceeds the threshold, the comparator will generate a spike as output and the control block will work to reset the memristor into high resistance state. We adopted the threshold memristor model described in the report.^[^ ^37^ ^]^ The SPICE simulation results are shown in Figure 7e,f with separate threshold values of 0.25 and 0.5, respectively. The integration part shows the capacitor begins to charge with coming pulses but does not exceed memristor threshold voltage. The neuron can adjust its sensitivity to incoming inputs based on external stimuli with humidity. The adaptability can be harnessed to implement and enhance the spike encoding scheme as shown in Figure 7g. We utilize the threshold encoding on the MNIST dataset, which converts the image pixel value into spike trains when the integrated input reaches a certain threshold. With different threshold values, the spike firing rate varies along with information density. A spike neural network (SNN) with a 784‐200‐10 architecture is utilized for further classification. The classification result is shown in Figure 7h. The neuron with a threshold value of 0.5 achieves an accuracy of 88.42% while the one with a threshold value of 1.0 results in a lower accuracy of 80.35%. The result indicates that our memristor based neuron has different encoding abilities, which can potentially work as humidity adaptive neuron in bio‐mimic systems.

**Figure 8:**
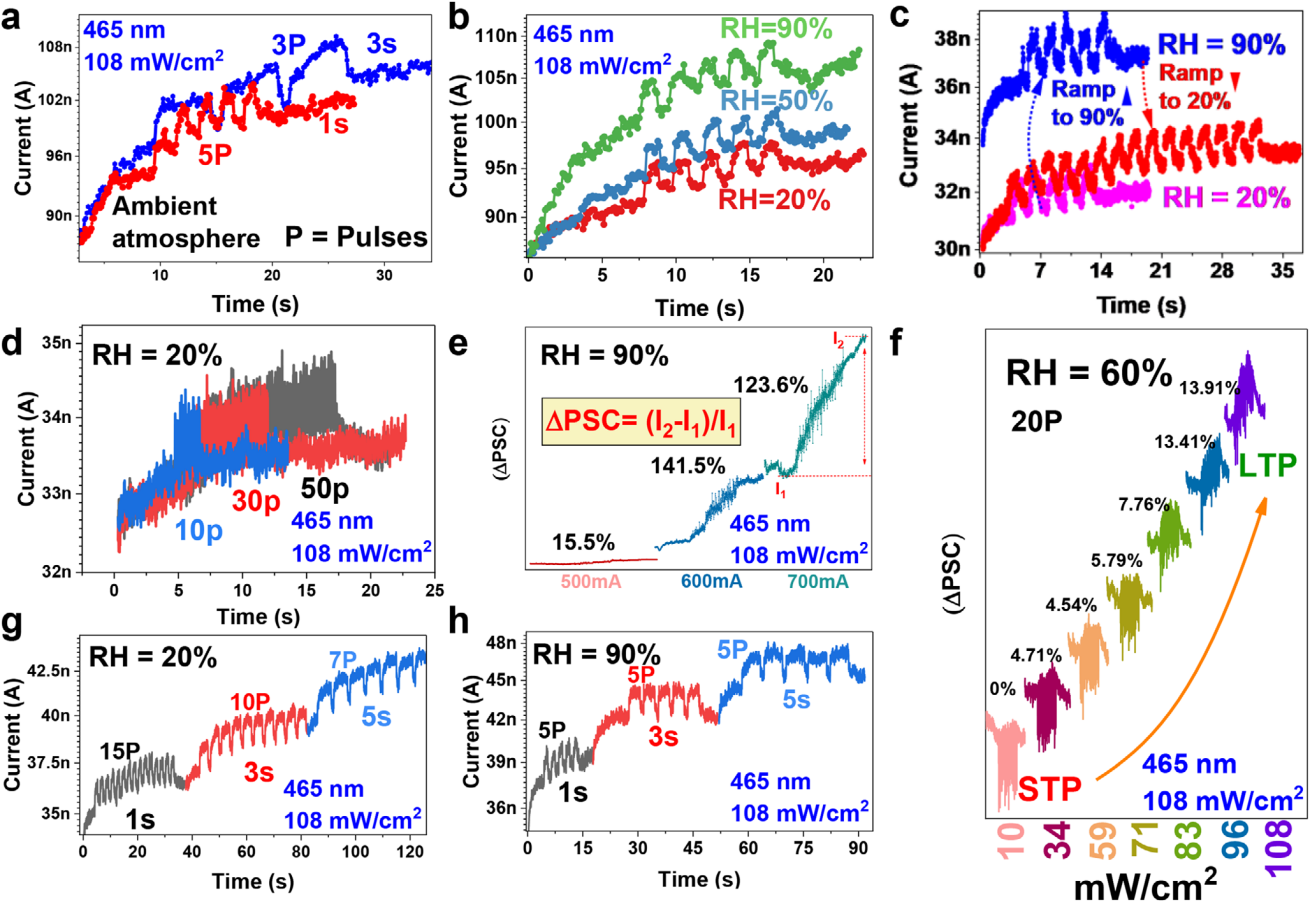
Humidity mediated light‐induced PSC using the 465 nm blue light a) in ambient atmosphere (laser current: 900 mA) b) under RH of 20, 50, and 90% c) after ramping the RH from 20% up to 90% and ramping down to 20% d) with pulse and interval width of 0.1s for 10, 30, and 50 pulses at RH = 20% (e based on increasing the laser current from 10 – 108 mW cm^−2^ at RH = 90% (pulse and interval width = 0.1s). f) STP to LTP based on increasing the laser power from 10 – 108 mW cm^−2^ at RH = 60% (pulse and interval width = 0.1s for 20 pulses). g) based on increasing the pulse width (1s, 3s, and 5s) at RH = 20% h) based on increasing the pulse width (1s, 3s, and 5s) at RH = 90%.

The light induced synaptic behavior of the MoWS_2_‐VO*_x_* heterostructure were found to be uniquely tunable and versatile under different RH levels. The multi‐variable analysis of both the light and the humidity engineering mode with synaptic learning characteristics showed the excellent multimodality of the device. **Figure** **8**a shows the synaptic learning at room level humidity and the device currents experienced an increase in the PSC when 1s, and 3s pulses were applied with a pulse width of 1s (900 mA). Further, with increasing the humidity from 20% to 50% and 90%, the increase in the PSC was different at the same pulse width, and number of pulses was observed. The increase in current is obvious at higher humidity levels from the Figure 8b. Further, the humidity mediated light induced synaptic behavior was measured by ramping up the humidity from 20% to 90% and then ramping down the RH from 90% to 20% (Figure 8c). We observed a reversible nature and tunability of the device wherein the currents were suppressed to ≈ its original value (i.e., as observed for RH = 20%). The reversibility post RS switching was also seen in the I‐V characteristics (Figure 6b and Figure S18c, SI) as discussed earlier showing the reconfigurable nature of the device. Figure 8d shows the enhanced learning behavior after the number of pulses were increased from 10 to 30 and 50P (pulse and interval width = 0.1s) for measurements at RH = 20%. Figure S18b, SI shows that the PSC increased more significantly when the RH was set to 90%, indicating that the device was more sensitive to light at higher humidity levels. Figure 8e shows the increased PSC response when the laser power intensity was increased from 10– 59 mW cm^−2^ at RH = 90% and Figure 8f and Figure S18a, SI shows the STP to LTP transition when the laser power intensity was increased from 10–108 mW cm^−2^ at RH = 60% having a pulse / interval width of 0.1s for 20P and 10P, respectively. As the number of pulses were increased from 10 to 20P, the ΔPSC increased from 1 to 4.71% and 11.9 to 13.91 when the laser power intensity was 34 and 108 mW cm^−2^, respectively. This demonstrated the STP to LTP behavior based on the increase in the laser power intensity and number of pulses. The sudden and rapid increase in the PSC at RH = 90% as compared to the change in PSC at RH = 60% is clear. It is due to the combined effect of leakage currents and other factors. The humidity‐mediated, light‐tunable optoelectronic dynamics in VO_x_/MoWS_2_ heterojunction are strongly influenced by both interfacial and bulk trap sites. Water molecules can indirectly generate these trap states within the oxide by reacting with oxide ions to produce hydroxyl ions and protons, thereby introducing additional electronic traps. In dry conditions, the surface states may induce band bending, which can hinder the separation of photo‐generated charge carriers and promote recombination. While in humid conditions, water molecules can potentially passivate these surface states, leading to improved charge separation and transport. This phenomenon corroborates with the switching mechanism governed by O²⁻ ion migration across the VO_x_/MoWS₂ interfacial barrier. The observed humidity adaptive behavior stems from the reduced mobility of O²⁻ ions under dehydrated conditions, as water molecules typically facilitate ion diffusion by providing protons and oxygen within the active layer. Nonetheless, it is hard to anticipate how humidity itself contributes to tuning photonic excitation due to light pulsing, but its effect on the electrical characteristics is clear. It is a complex phenomenon where the light intensity, surface reflections, and light absorption could be altered by the drastic changes in the humidity. This in turn influences the light – material interaction at the photonic level. Figure 8 g, h shows that the increase in PSC at RH of 20% was significantly lower than the response at RH of 90%. The increase in PSC at the RH of 20% (15,10, and 5P of 1, 3, and 5s, respectively) is lower than that observed at RH of 90% (5P each of 1, 3, and 5s). Increased illumination time or a higher number of light pulses enhanced the formation of charge trapping centers for photogenerated charge carriers (electrons and holes). This resulted in effective storage of these carriers, contributing to a higher PSC at a slower decay rate. In addition, increasing the light intensity increased the optical excitation energies thereby increasing the PSC. Figure S18d,e, SI furthers demonstrates the changes in the PSC with increase in the pulse width from 1s → 3s → 5s using 465 nm laser having 101 mW cm^−2^ laser power intensity at RH = 60% and the laser power intensity from 10 mW cm^−2^ → 101 mW cm^−2^ → 108 mW cm^−2^ at RH = 60%.

VO_2_ is a very promising candidate but readily undergoes degradation in the environmental conditions. VO*_x_* thin films with VO_2_ as the dominating phase tend to degrade in environmental conditions because VO_2_ has a greater tendency of being slowly oxidized to V_2_O_5_ under room temperature condition.^[^ ^38^ ^]^ It has been previously shown that, if VO_2_ is wrapped in the amorphous V_2_O_5_ matrix, it would act as a barrier to reduce the oxygen infusion into the film matrix thereby stabilizing the overall VO*_x_* layer. This work employed a similar approach to overcome the issue of degradation in vanadium oxide films by developing VO*_x_* films with a V_2_O_5_ dominant phase. The electrical properties of the fabricated devices were evaluated over a prolonged interval of 6 months. The results indicate no observable degradation in their electrical performance (Figure S19, SI), which ensured the stability of the fabricated heterojunction memristor devices. Moreover, in this study, Mo_0.5_W_0.5_S_2_ is utilized which is less prone to photo‐oxidation than WS_2,_ MoS_2,_ and Mo_0.25_W_0.75_S_2,_ as previously reported.^[^ ^39^ ^]^ Furthermore, the MoWS_2_ layer is encapsulated with the Cu electrode (Pt/VO*_x_*/MoWS_2_/Cu). HRTEM results (Figure S20, SI) did not reveal any significant oxidation of the MoWS_2_ layer. TFor a better understanding, the HRTEM analysis was conducted on the same device that had been exposed to humidity and light during the prolonged measurements (6 months), while the electrical properties of the fabricated devices were also assessed after the extended interval of 6 months. The results indicate no observable degradation in their electrical performance (Figure S20a, SI), which confirms the stability of the fabricated heterojunction memristor devices. Optical measurements on the devices were also performed after exposing them to the environment (including light and humidity) for 6 months. The results showed no significant effect of photo‐oxidation on the optical properties of the device as observed from Figure S20b–e, S.

**Figure 9:**
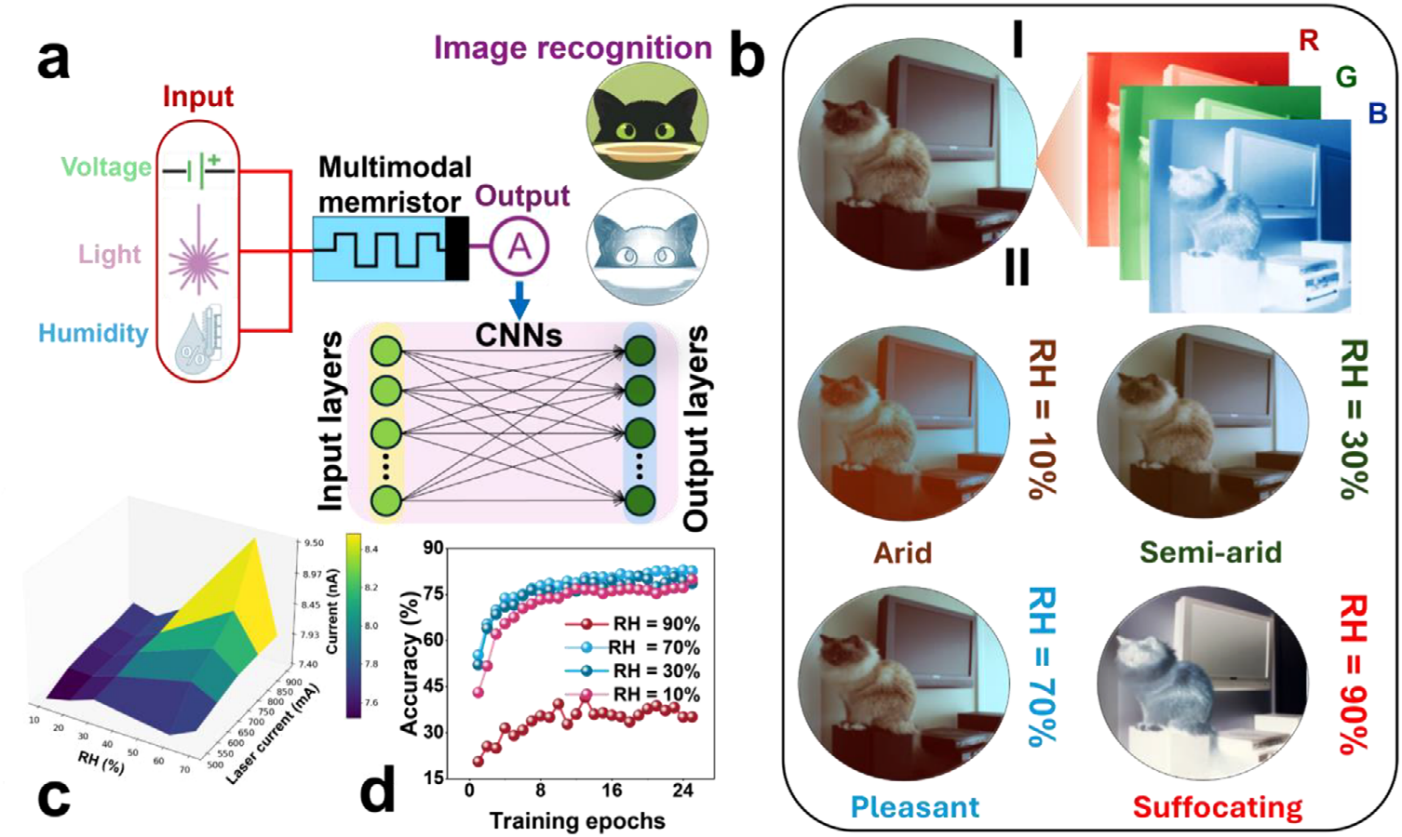
a) Image recognition in the light modulated and humidity mediated neuromorphic visual system demonstrating how humidity affects vision clarity. b) Sample figure visualization with RGB channels and sample visualization at different humidity levels. c) Current response under different RH levels and laser currents. d) Training accuracy V/s epochs for different RH levels. The cat image in (b) is adapted from the Oxford‐IIIT Pet Dataset (https://www.robots.ox.ac.uk/~vgg/data/pets/) under the terms of the CC‐BY Creative Commons Attribution 4.0 International license (https://creativecommons.org/licenses/by/4.0).

**Figure** **9**a shows the schematic illustration of the humidity mediated and light modulated MoWS_2_/VO*_x_* heterojunction memristor demonstrating the simultaneous effect of humidity and light on the vision clarity. As shown in Figure 9 (b_I_), the color image sample selected from Oxford‐IIIT Pet Dataset was sampled into R, G, and B channels. In the experiment, photocurrent increased more significantly with higher light intensities and humidity levels. Figure 9c shows the current response under varying humidity levels and power, where it shows a peak at high humidity and light intensity. We evaluated and visualized the image with humidity levels of 10%, 30%, 70%, and 90% at a laser current of 700 mA, as shown in Figure 9 (b_II_). The visualization results at 30% and 70% RH produce clearer, more visually comfortable images with more natural colors compared to those at 10% and 90% RH. We assessed the humidity sensing capability using the CNN on the Oxford‐IIIT Pet Dataset, with different humidity levels applied as a data augmentation preprocessing method. Figure 9d shows the training results improved with the number of epochs. The training results demonstrated a steady increase in accuracy, with moderate and human comfortable humidity levels contributing to a greater accuracy.

**Figure 10:**
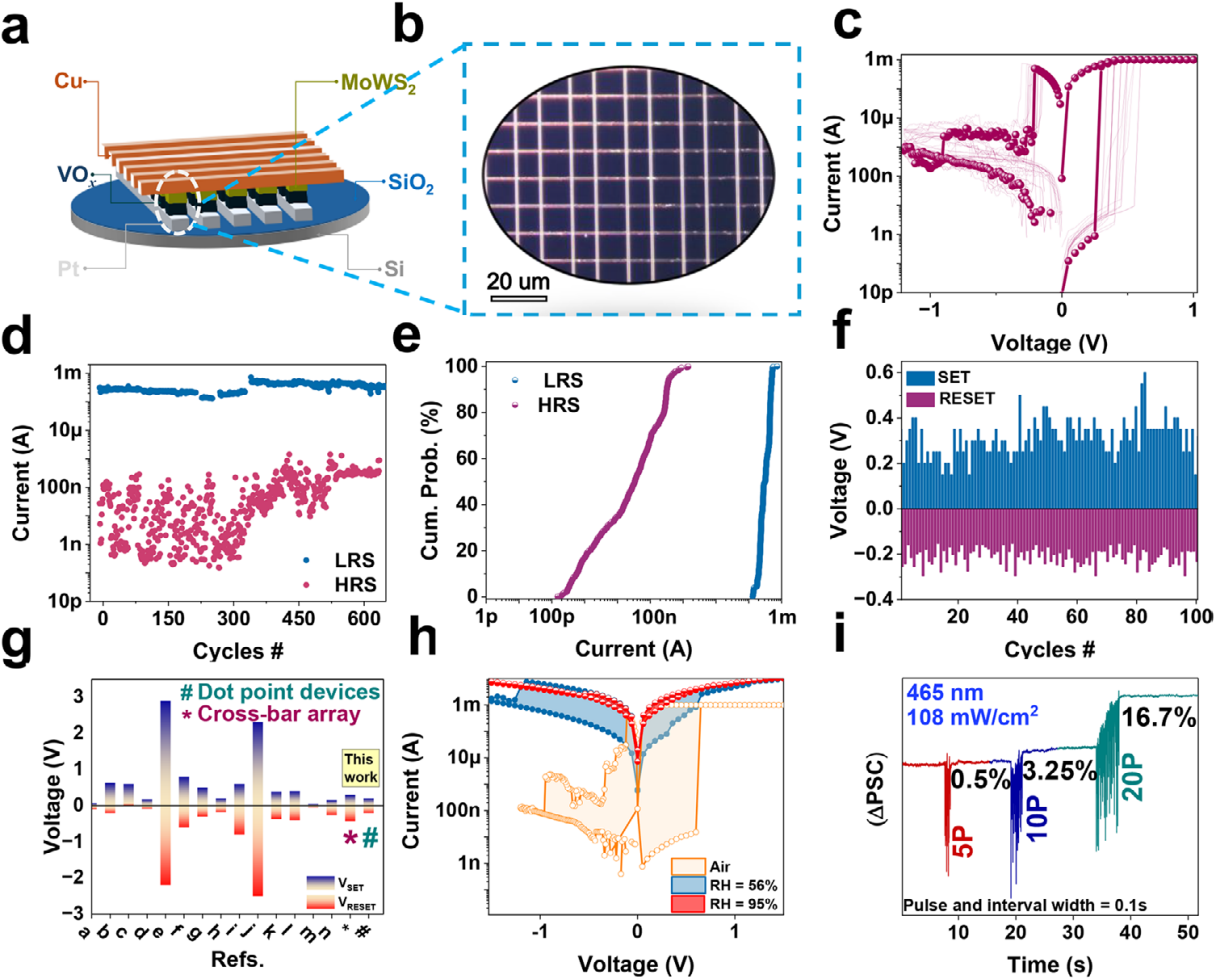
a) Schematic representation of the memristor crossbar array, b) an inset displaying the FESEM image of an individual device. c) Current‐voltage (I–V) characteristics of a randomly selected memristor device from the crossbar array, demonstrating consistency with previously studied single devices in terms of memory window and operating voltage. d) Cycle‐to‐cycle endurance of randomly selected devices measured up to 600 cycles, with read operations performed at 0.05 V. e) Cumulative probability distribution of the LRS and HRS during endurance cycles. e) Cycle‐to‐cycle variability in resistive switching voltages (V_SET_ and V_RESET_) analyzed over 100 DC cycles. f) Comparative analysis of the SET/RESET voltages across different studies. g) Comparative analysis of the SET/RESET voltages.^[^ ^40^ ^]^ h) Humidity‐responsive I‐V characteristics exhibit comparable trends under crossbar conditions. i) Optically stimulated response under illumination with a 465 nm light source.

To confirm that the device performance can be reproduced and is reliable at large‐scale fabrication levels, we fabricated crossbar arrays of the heterojunction device. As depicted in **Figure** **10**a, a crossbar array (an optical image of which is shown in Figure 10b) of Cu/MoWS_2_/VO_x_/Pt devices with a 10 µm^2^ device area was fabricated. Preliminary results from this array were investigated to assess its compatibility with large‐scale fabrication. Figure S21, SIpresents a schematic illustration of the steps involved in the fabrication process of the MoWS_2_/VO*_x_* heterojunction‐based crossbar array. Furthermore, as shown in Figure 10c, randomly selected and measured devices exhibit resistive switching behavior similar to that observed in the previous batch of dot‐point devices fabricated at a smaller scale. Figure 10d illustrates the endurance performance measured over 600 cycles, demonstrating a similarly large memory window, as evidenced by the I_on_/I_off_ ratio, compared to the dot point devices. Additionally, Figure 10e presents the cumulative probability distributions of the LRS and HRS. The cycle‐to‐cycle variability in switching voltages was analyzed over 100 DC cycles was found to be low, as depicted in Figure 10f. The mean values of V_SET_ and V_RESET_ were found to be 0.290 and −0.355 V, respectively. Figure 10g presents a benchmark of our dot‐point devices (#) and devices in the crossbar array's (*) switching voltages against various other devices. The values for the * and # represent the V_mean_ from 100 switching cycles. Additionally, both humidity‐dependent and optically stimulated resistive switching have been demonstrated, as illustrated in Figure 10h,i.

While large‐scale fabrication of the memristor devices shows potential scalability, we observed slightly larger variability in the RESET cycles compared to dot‐point devices due to non‐uniform thickness in the MoWS_2_ deposition process (Figures S22–S24, SI). This non‐uniformity, which is less pronounced at small scales, leads to variability in device performance across large arrays. To address this limitation, future work will focus on optimizing the MoWS_2_ deposition process to achieve a more uniform thickness across larger areas. Additionally, alternative deposition techniques should be investigated to improve reproducibility and reduce device‐to‐device variability. These improvements will be critical for ensuring the reliable performance of large‐scale memristor arrays for practical applications.

## 3 Conclusion

In summary, we have developed a heterojunction multifunctional memristor device configured as Cu/MoWS_2_/VO*_x_*/Pt. This device demonstrates a remarkable on/off ratio of 10^8^, coupled with non‐volatile state retention validated for durations extending up to 40 000 s. With such a high on/off ratio, the device remarkably exhibited potentiation and depression behaviors analogous to biological neurons. Furthermore, the integration of this device into multisensory systems such as electrical, optical and moisture stimulation presents a promising avenue for future research. Notably, the device exhibits optically stimulated reset properties when exposed to light sources with wavelengths of 532, and 635 nm. In addition to its optical sensitivity, the device shows humidity responsiveness across a range of 5% to 95% RH, resulting in more pronounced resistance switching and lower operational voltages. Device shows reconfigurable modulation in both memory and synaptic functionalities, providing tunable conductance modulation capabilities that emulate synaptic transmission in biological neurons as well as shows potential in respiratory detection module for healthcare application along with image recognition. In addition, the device can be operated and controlled via electrical stimulation, optical stimulation and moisture stimulation, independently as well as simultaneously using a 465 nm light source. Furthermore, humidity sensing devices depicts potential in the respiratory detection module. To further assess its classification capabilities, we employed a SNN architecture utilizing threshold encoding on the MNIST dataset (based on humidity sensing data) as well as CNN based architecture for Oxford‐IIIT Pet Dataset image visualization (based on humidity mediated optical synaptic plasticity data). This configuration achieved a compelling accuracy of 88.42% at a threshold voltage of 0.5 V and 83.30% at 700 mA laser current, respectively. The findings regarding this multimodal functionality indicates the potential of this device as a novel platform for the development of multi‐stimuli compatible artificial sensory neurons, with significant implications for non‐contact human–computer interaction in intelligent systems.

## 4 Experimental Section

### Device Fabrication

Figure S1, SI illustrates the fabrication process of the MoWS_2_/VO*_x_* device. A Si wafer was used as a substrate for the device fabrication. First the native oxide of Si was etched away using buffer oxide etchant (BOE) for 3 min and then rinsed with deionized water. The Si wafer was then dried using a N_2_ gun. The fabrication involved deposition of 250 nm SiO_2_ using plasma enhanced chemical vapor deposition (PECVD) at 250 °C. A 10 nm thick Ti layer (adhesion layer) was deposited using RF sputtering. Further, a 100 nm thick Pt layer was deposited via RF sputtering as the bottom electrode. In the later step, ≈80 nm amorphous vanadium oxide (VO*_x_*) was deposited utilizing VO_2_ target via reactive sputtering. aThe sputtering pressure was set to 4mTorr, while the chamber pressure was maintained at 20mTorr. Afterwards, MoWS_2_ flakes were drop casted and the wafer was dried in ambient conditions. Finally, a 65 nm thick layer of Cu was deposited through shadow mask using RF sputtering completing the Cu/MoWS_2_/VO*_x_*/Pt device stack. The fabrication process for the crossbar array devices is illustrated in Figure S21, SI.

### Characterization

The cross‐sectional structure, and the layer thickness were characterized using high‐resolution transmission electron microscopy (HRTEM) on a Titan Cs Probe at 400 keV. The elemental composition was acquired in the STEM mode using energy‐dispersive spectroscopy (EDS). The TEM lamella was prepared using the focused ion beam scanning electron microscopy technique (FIB‐SEM) on a Helios G4 FIB/SEM (Thermo Fisher Scientific). X‐ray photoelectron spectroscopy (XPS) data were obtained using a Kratos Axis Supra instrument equipped with a monochromatic Al Kα X‐ray source (h*ν* = 1486.6 eV) operating at a power of 75 W and under UHV conditions in the range of ≈10^−9^ mbar. All spectra were recorded in hybrid mode, using electrostatic and magnetic lenses and an aperture slot of 300 µm × 700 µm. The wide and high‐resolution spectra were acquired at fixed analyzer pass energies of 80 and 20 eV, respectively. The sample was mounted in a floating mode to avoid differential charging. The following aspects should be considered to obtain accurate peak fitting results. First, it is necessary to take into account the O 1s photoelectron line and use Shirley background for the whole region of V 2p + O 1s prior to start the fitting process.^[^ ^21a^ ^]^ Indeed, O 1s and V 2p core levels are close to each other so that excluding O 1s from the fitting will have strong influence on the background underneath the V 2p signal.^[^ ^21a^ ^]^ Also, it is important to adjust the calibration properly because, in case of vanadium oxide compounds, it has been demonstrated that using oxygen for referencing the binding energy scale by setting it at 530 eV provides more accurate data compared to using carbon which generates overestimated binding energy values.^[^ ^21a^ ^]^ Last, some constrains have to be used, in particular, the ratio between V 2p_3/2_ and V 2p_1/2_ areas should be fixed to 2:1. Moreover, the component separation “doublet splitting” between V 2p_3/2_ and V 2p_1/2_ core levels has to be in the range of 7.3 to 7.6 eV.^[^ ^29a,b^ ^]^ X‐ray diffraction (XRD) spectra were recorded using a tabletop Xray diffraction instrument: Bruker D2 phaser equipped with a Cu tube source (λ = 1.54184 Å) at 30 kV. The surface roughness and the topography of the MoWS_2_/VO*_x_* heterostructure was obtained using atomic force microscopy (Bruker Dimension icon SPM). Further, the transmittance of the VO*_x_*, MoWS_2_, and MoWS_2_/VO*_x_* thin films was obtained using UV–visible spectroscopy (UV–vis‐NIR Lambda 950). Secondary ion mass spectroscopy (Hiden) was used for the elemental identification of commercially procured MoWS_2_.

### Electrical Measurements

The electrical characteristics were measured using the semiconductor parameter analyzer (Agilent B1500A). The optoelectronic measurements were carried out using 456, 532, and 635 nm lasers (Shanghai Dream Lasers Technology), and the light pulses were controlled using the electronic shutter controller (Newport).

### Humidity Sensing Measurements

The humidity sensing measurements were carried using an integrated multisensory setup having precision humidity control system (Nextron: HCS‐2 M). The device was mounted inside the sensing chamber of the micro probe station (Nextron) at a temperature of 25 °C. The humidity ramping time was set to 2 min and N_2_ gas pressure was set to 2 mbar. The electrical measurements were carried out using Keithley (4200 SCS).

## Conflict of Interest

The authors declare no conflict of interest.

## Author Contributions

Abdul Momin Syed and Nazek El‐Atab conceived this work. Abdul Momin Syed developed this study, including the fabrication, characterization, and testing of the devices, as well as the manuscript preparation/writing. Dhananjay Kumbhar contributed to the electrical measurements and manuscript writing. Hanrui Li assisted in the development of the humidity‐adaptive neuron and the humidity‐affected visual perception application. Manoj Kumar Rajhbar performed UV–vis. spectroscopy measurement and designed the mask for the cross‐bar array. Dayanand and Pratibha engaged in a discussion regarding electrical measurements and reviewing the manuscript. Mohamed ben Hassine supported imaging and indexing the FFT of MoWS_2_. Nimer Wehbe characterized and analyzed the HRXPS results of the VO*_x_* thin film. Nazek El‐Atab provided guidance on the project, supervised the research, and contributed funding and critical review.

## Supporting information



Supporting Information

## Data Availability

The data that support the findings of this study are available from the corresponding author upon reasonable request.
