# Supplementary material for: A Multimodal Humidity Adaptive Optical Neuron Based on a MoWS2/VO x Heterojunction for Vision and Respiratory Functions
Source: Adv Mater. 2025 Apr 29;37(27):2417793. doi: 10.1002/adma.202417793 (PMC12243701; doi:10.1002/adma.202417793)
Supplement: Supplementary file 1 — Supporting Information [file ADMA-37-2417793-s001.docx]

**Supplementary Information**

**A Multimodal Humidity Adaptive Optical Neuron Based on a MoWS_2_/VO*_x_* Heterojunction for Vision and Respiratory Functions**

Abdul Momin Syed^1^, Dhananjay D. Kumbhar^1^, Hanrui Li^1^, Manoj Kumar Rajbhar^1^, Dayanand Kumar^1^, Pratibha Pal^1^, Nimer Wehbe^2^, Mohamed bin Hassine^2^, and Nazek El-Atab^1,^*

^1^Smart, Advanced Memory Devices and Applications (SAMA) Laboratory, Electrical and Computer Engineering, Computer Electrical Mathematical Science and Engineering, King Abdullah University of Science and Technology (KAUST), 23955-6900, Thuwal, Saudi Arabia

^2^The Imaging and Characterization Core Lab, KAUST.

*Corresponding Author: Nazek El-Atab ([nazek.elatab@kaust.edu.sa](mailto:nazek.elatab@kaust.edu.sa))


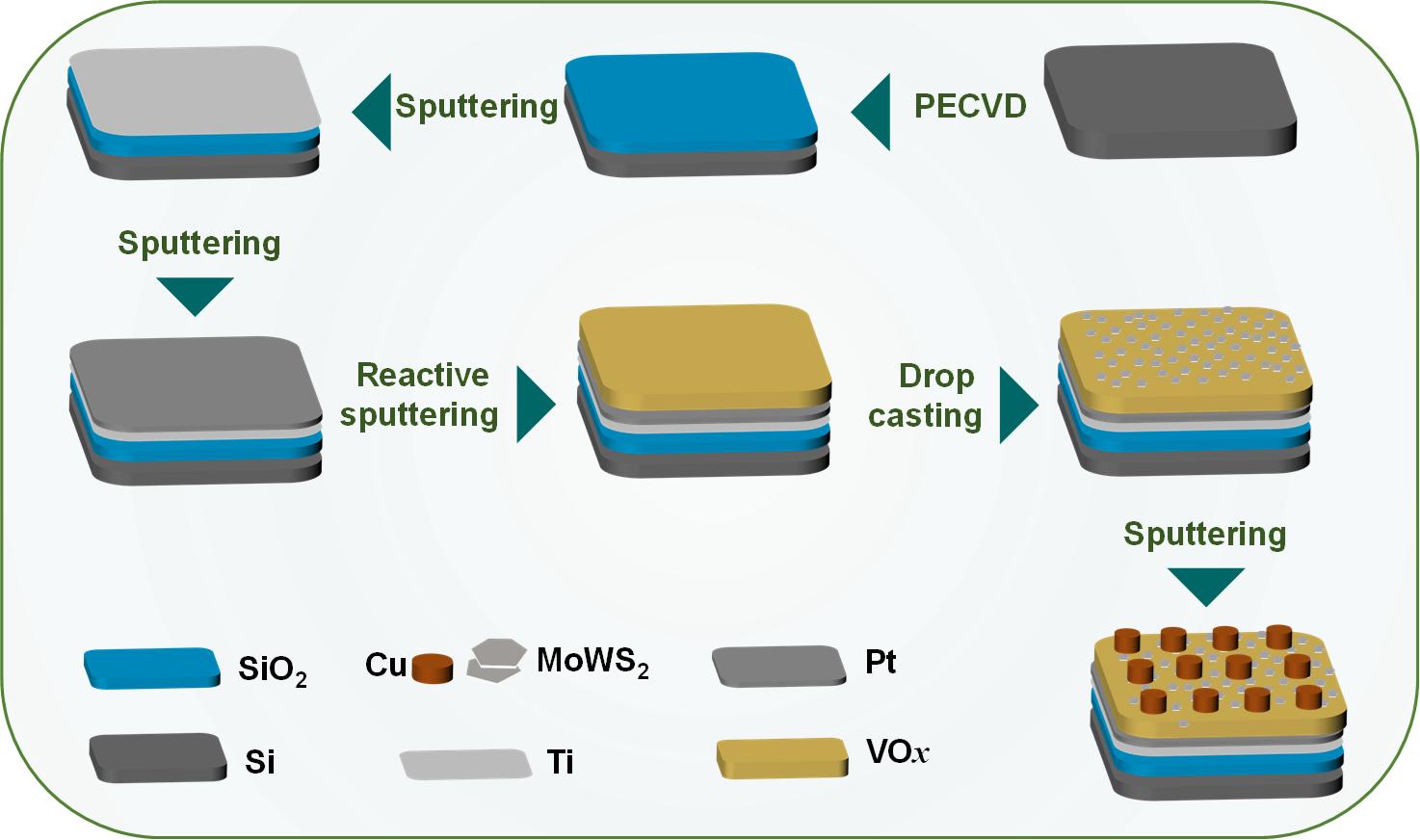


**Fig. S1** Schematic diagram of the fabrication process flow of Cu/MoWS_2_/VO*_x_*/Pt memristor device

**Fig. S2** (a) EDS spectrum of MoWS_2_ flakes (b) HRSTEM and FFT indexing of MoWS_2_ structure.


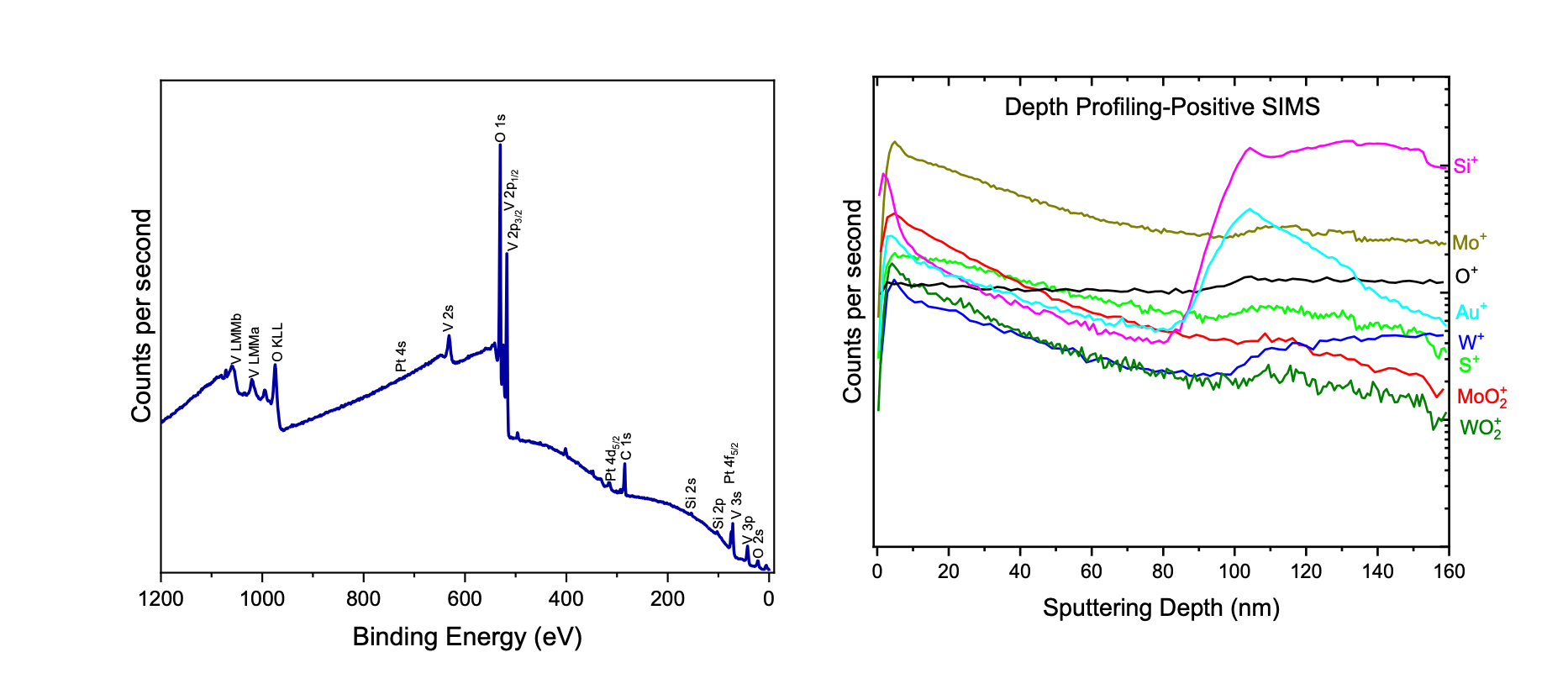


**b**

**a**

**Fig. S3** (a) Survey HRXPS of VO*_x_* and (b) SIMS profile of MoWS_2_ thin film.

**b**

**a**


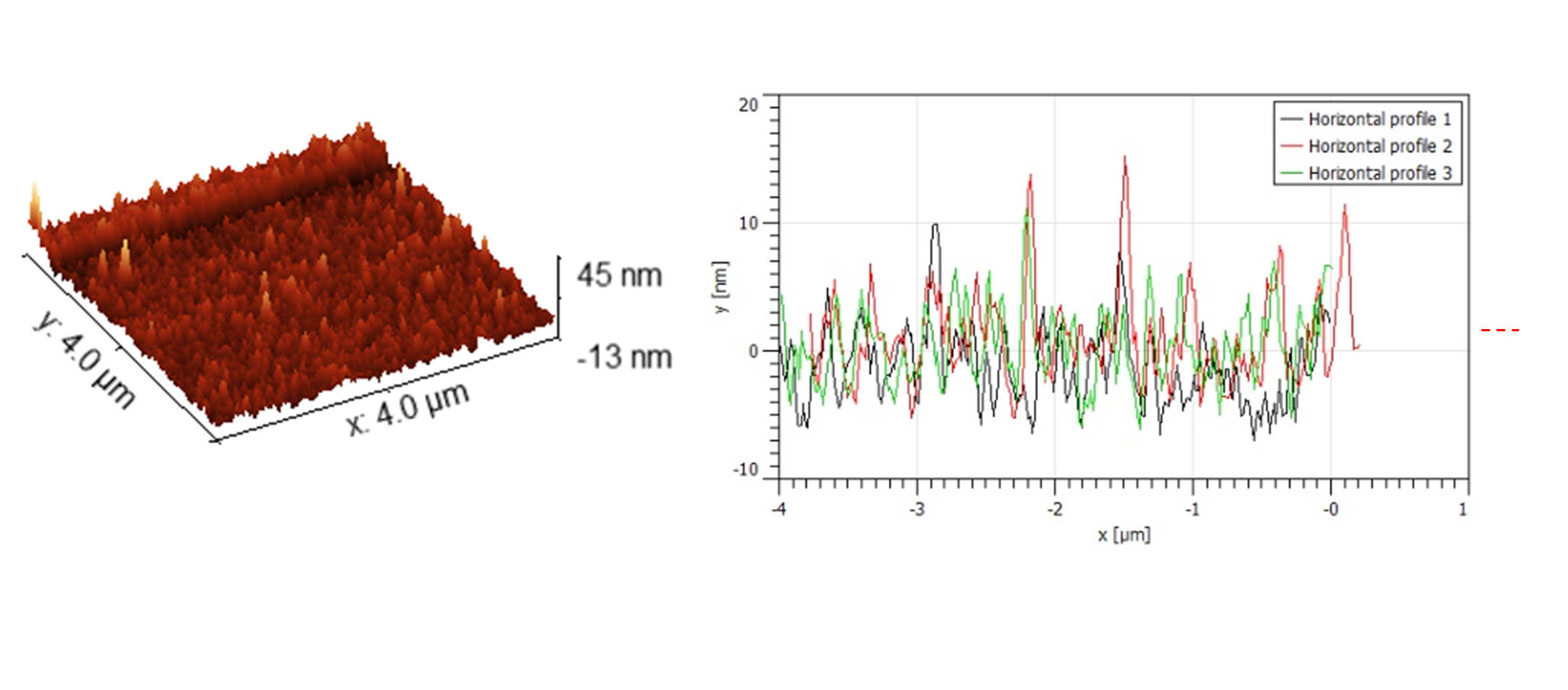


**Fig. S4** (a) 3D AFM image and (b) line scan across an arbitrary area of the of the MoWS_2_/VO*_x_* heterojunction.


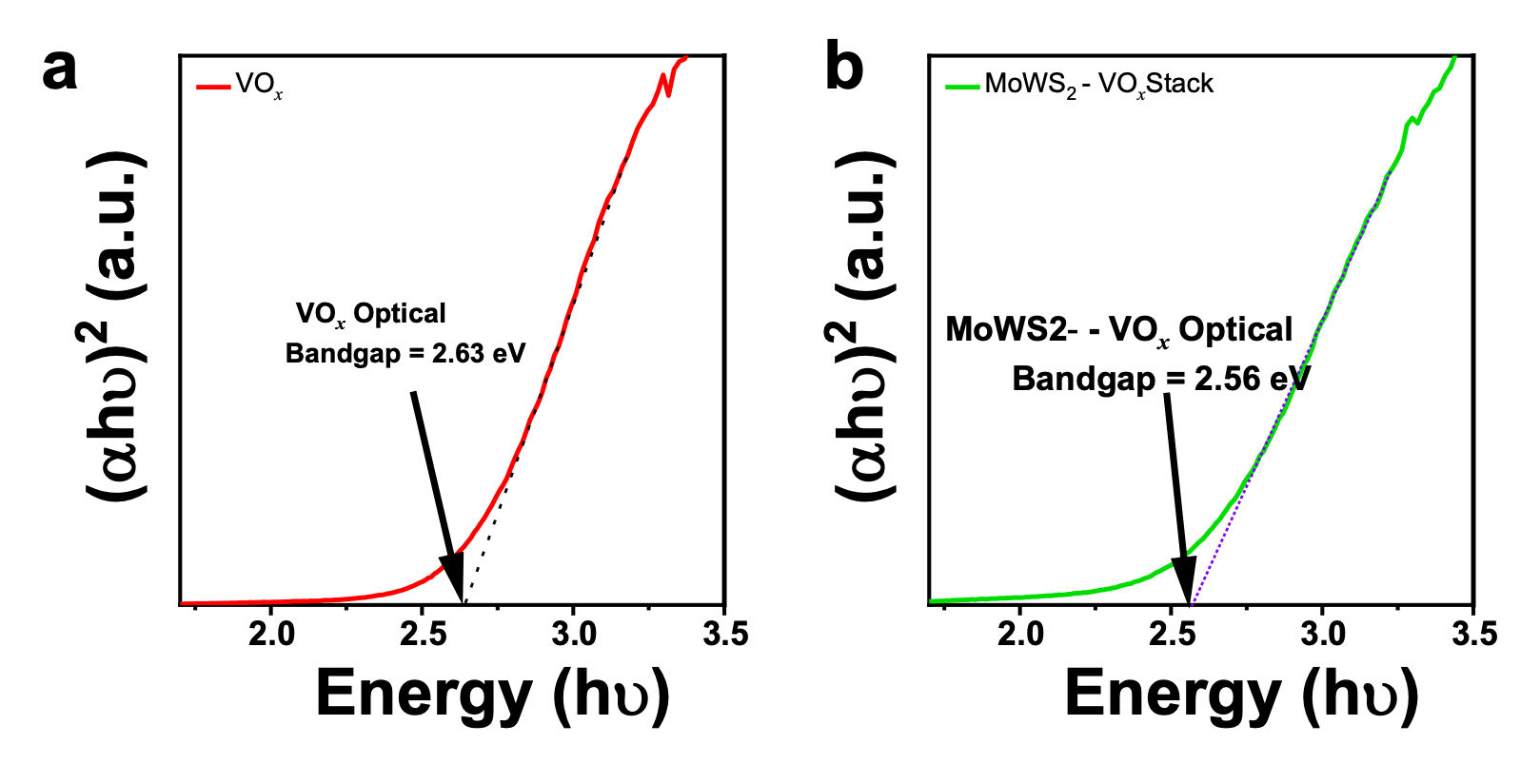


**Fig. S5** Optical band gap of (a) VO­*x* and (b)MoWS­_2_ thin film.

**Fig. S6** I-V curves of electro-forming of 7 arbitrary devices.

**Table 1. Table of Comparison: Key Switching Parameters for Multimodal Memristor devices**

| Functional 2D material | Switching voltage | On/Off ratio | Off current | Retention | CC (A) | Synapse | Modes | Ref. |
| --- | --- | --- | --- | --- | --- | --- | --- | --- |
| Pt/HfO*_x_*/GO/Ti | -2/~5.5V | 10^4^ | 10^-8^ | No | 10^-4^ | P/D | 2 (E, H) | ^[1]^ |
| Ag/c-YY NW/Ag | 0.5/-0.5 | 10^4^ | 10^-11^ | - | 10^-6^ | - | 2 (E, H) | ^[2]^ |
| Al/Ni–Al LDHs/ITO | -5/5 | 10^5^ | 10^-9^ | 10^4^ | - | - | 2 (E, H) | ^[3]^ |
| Pt/HfO_2_/W | -3/3 | 10^3^ | 10^-7^ | - | 10^-4^ | P/D | 2 (E, H) | ^[4]^ |
| Ag-VO_2_-Ag | -2/2 | 10^4^ | ~10^-8^ | - | - | - | 2 (E, H) | ^[5]^ |
| Au/CH_3_NH_3_PbI_3_/FTO | -2/1.5V | 10^3^ | 10^-5^ | 10^4^s | - | - | 2 (E, H) | ^[6]^ |
| Ag/ZnO NWs/Pt | 13V | 200 | 10^-7^ | - | 10^-5^ | - | 2 (E, H) | ^[7]^ |
| Gr/*α*-In_2_Se_3_/h-BN/Cr-Au | -4/7.3V | 10^9^ | 10^-14^ | - | 10^-4^ | - | E | ^[8]^ |
| ITO/Mxene-ZnO/Al | -0.5/1.2V | 10^4^ | 10^-7^ | 10^4^ | 10^-1^ | Humidity and optical mediated P/D | 3 (E, H, & T) | ^[9]^ |
| Ag/CIGSe/Mo | -2/2 | ~1.7x  10^3^ | 10^-7^ | 4500s | 10^-2^ | P/D | 3  (E, H, & T) | ^[10]^ |
| Cu/MoWS_2_/VO*_x_*/Pt | **-0.2/0.2** | **10^8^** | **10^-12^** | **4x10^4^** | **10^-3^** | **P/D, humidity adaptive neuron, and humidity mediated optical synapse** | **3 (E, H, & O)** | **This work** |

(E= Electric, H= Humidity, O = Optical, and T = Temperature, P = potentiation, D =depression.)


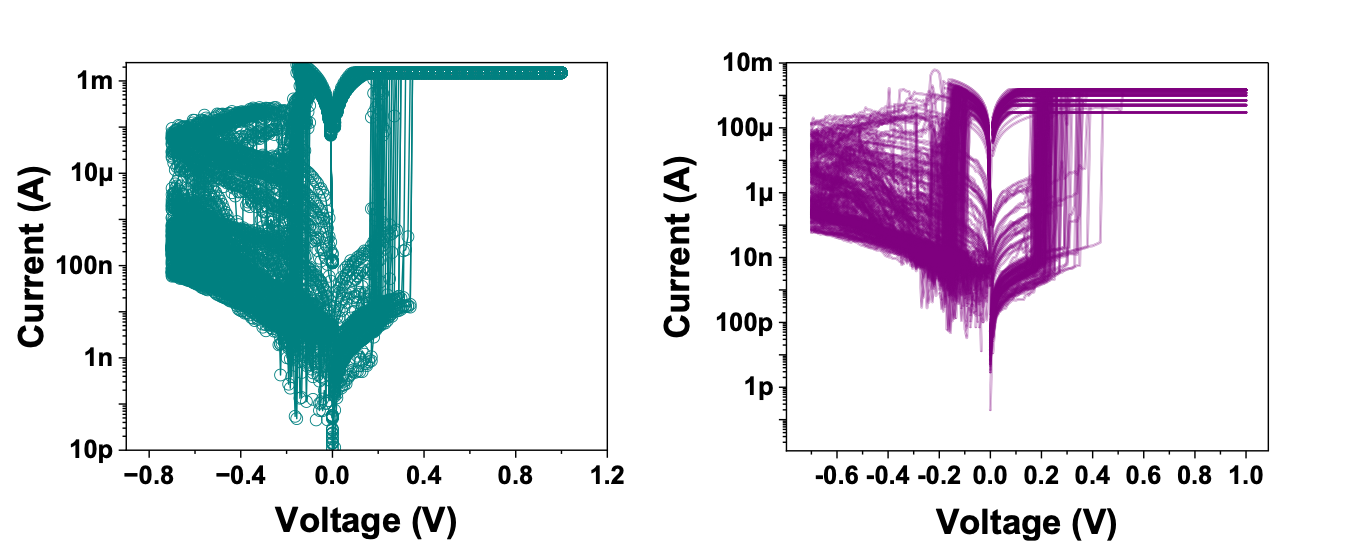


**a**

**b**

**Fig. S7 (a)** This study investigates the DC cyclic reliability of devices under varying compliance currents (CC) and (b) shows the endurance testing of the devices that was recorded at 0.1 V over 140 consecutive DC cycles.

**Fig. S8 (a)** I-V curves of Cu/MoWS_2_/Pt device with an On/Off ratio of 10^6^. (b) Comparison of the On/Off ratio of different devices.

**Fig. S9** Cyclic variations in switching parameters, such as V_SET_ and V_RESET_, were analyzed to assess the stability and consistency of device performance.

**Fig. S10** The distribution of Low Resistance State and High Resistance State current levels is presented in histogram form.


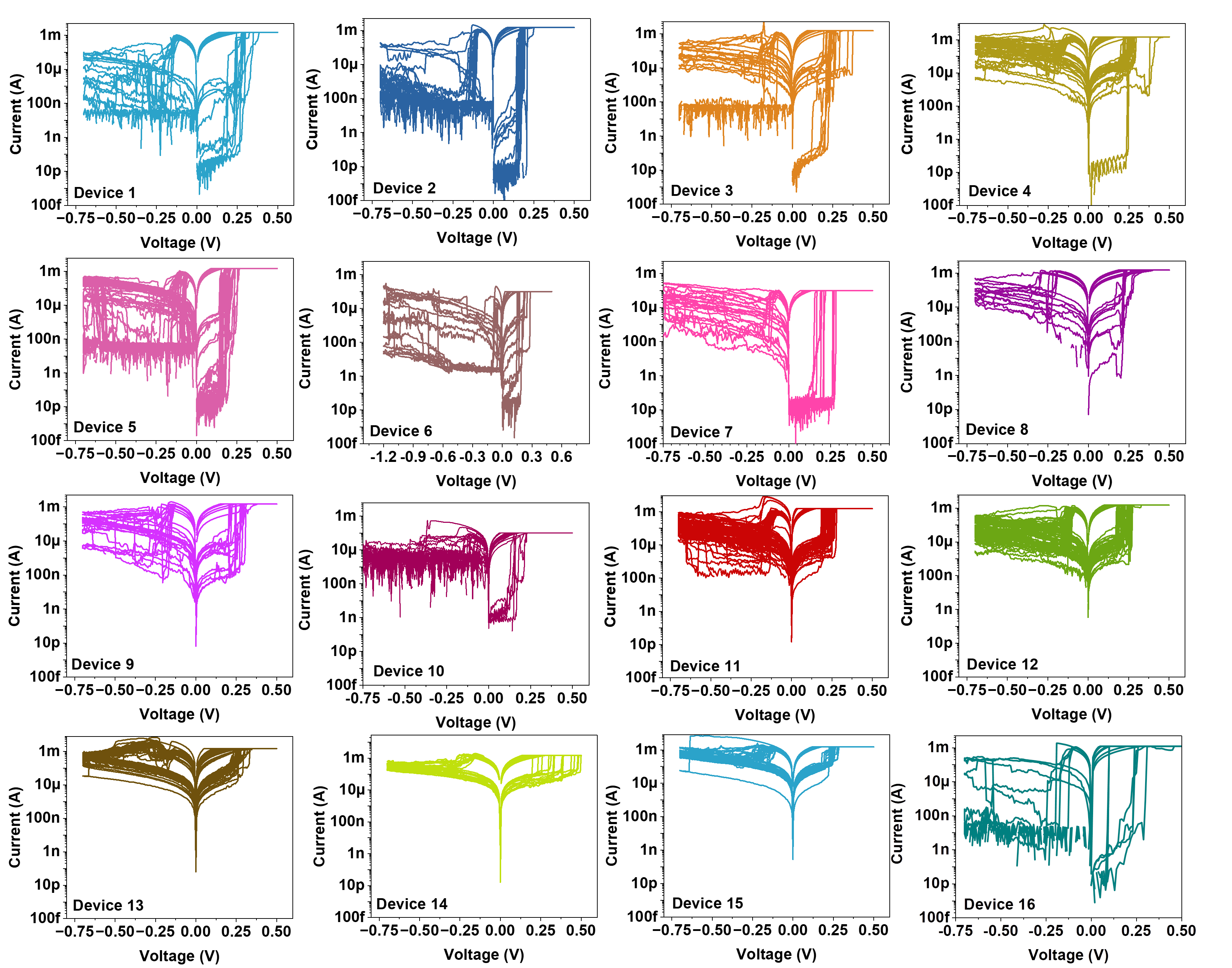


**Fig. S11** I-V characteristic of 16 arbitrarily chosen dot - point devices.

**Fig. S12** (a) Device to device variability across 16 arbitrarily chosen devices. (b) histogram distribution and (c) Cumulative probability of these 16 devices.

**Fig. S13** An illustrated overview of a plausible resistive switching mechanism is presented, detailing the transition from the pristine state to Low Resistance State (LRS) and High Resistance State (HRS) during the forming process. The diagram highlights potential filamentary contributions from copper (Cu), oxygen vacancies, and sulfur vacancies, elucidating their roles in the switching behavior and the formation of conductive pathways.


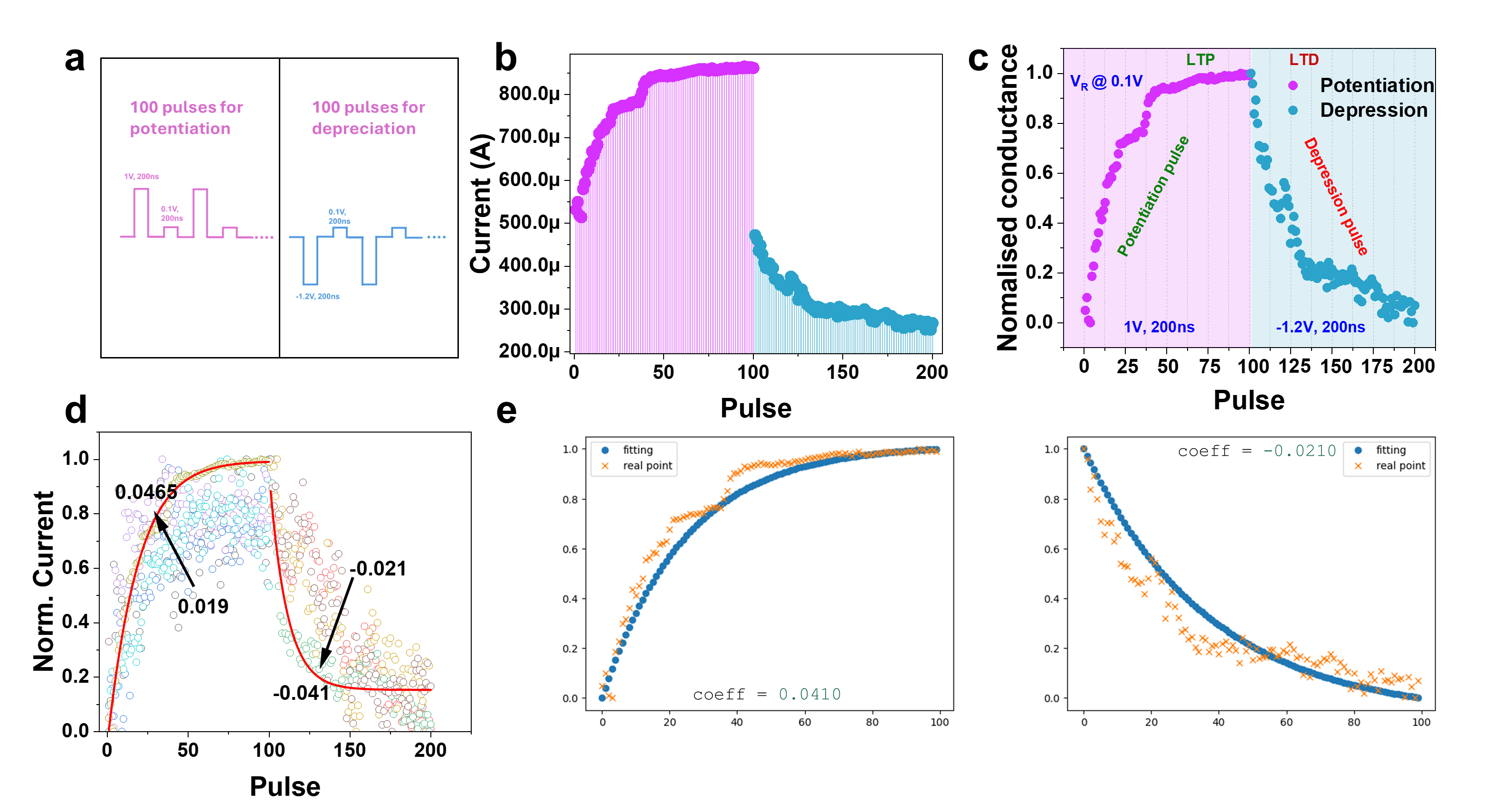


**Figure S14. (a)** Pulsing scheme for electrical potentiation and depression measurements. **(b)** Recorded potentiation (P) and depression (D) behaviors in the form of current at 0.1 V. **(c)** Normalized potentiation and depression responses scaled from 0 to 1. **(d)** Calculated non-linearity coefficients for both potentiation and depression values. **(e)** Depiction of the fitting of the ideal curve versus the actual measured curve during non-linearity fitting.


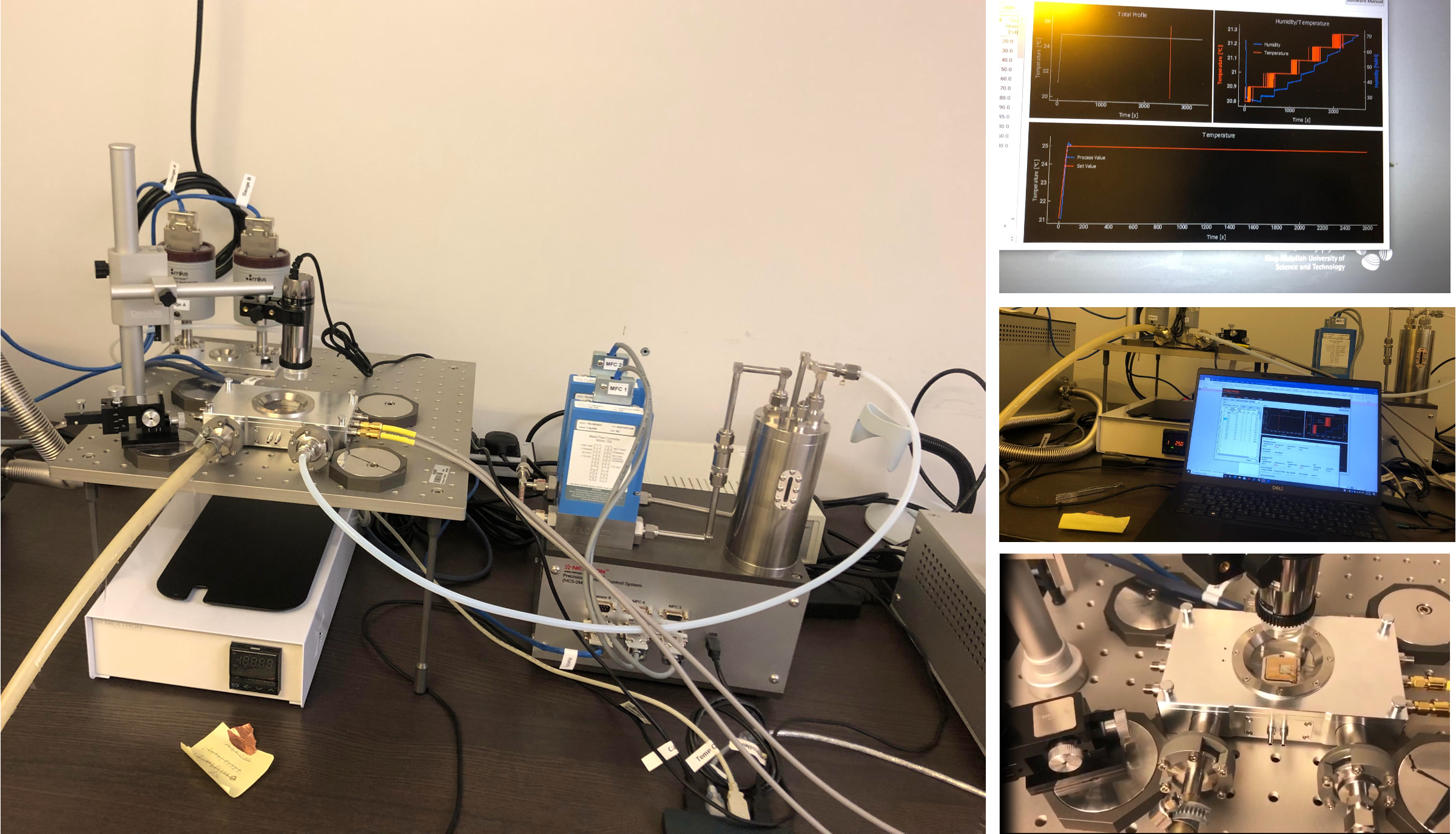


**Fig. S15** Humidity sensing setup.

**Fig. S16** (a) I-V cycles at different RH levels in negative sweep mode **(b**) I-V curves measured in air, at RH = 95% and the recovery of the device at RH = 20% and 10 I-V curve cycles at RH = (b) 5% (c) 56% (d) 95%

**Fig. S17** Schematic showing the effect of increasing humidity on the conductive filament formed across the heterojunction.

**Fig. S18** (a) STM to LTM based on increasing the laser power from 500 – 900 mA at RH = 60% (pulse and interval width = 0.1s for 10 pulses). (b) PSC increased with pulse and interval width of 0.1s for 30 pulses of 465 nm laser at RH = 90% (c) Difference in the PSC for RH = 20 and 90% (pulse and interval width of 1s for 5 pulses 465 nm laser). Increased PSC increase based on the (d) pulse width from 1s 🡪 3s 🡪 5s using 465 nm laser having 101 mW/cm^2^ laser power intensity at RH = 60%. (e) laser power intensity from 10 mW/cm^2^ 🡪 101 mW/cm^2^ 🡪 108 mW/cm^2^ at RH = 60%.

**Fig. S19** I-V characteristics of the (a) unexposed device and devices exposed to light and humidity levels up to 95% (b) after 5 and (c) 6 months of exposure.

**Fig. S20** (a) STEM EDS analysis of the MoWS_2_ layer showing minimal photo-oxidation of the MoWS_2_ layer covered by the Cu top electrode acting as a passivation layer. Optical measurements of the heterojunction device after 6 months of exposure to light and humidity using 465 nm laser (b, c) when the device is in its natural state i.e., HRS and (d, e) after electrically programming (SET cycle) the device to LRS.

**Fig. S21** Schematic illustration of the steps involved in the fabrication process flow of the MoWS_2_/VO*_x_* heterojunction-based crossbar array.

 **Fig. S22** (a) The I-V curves (100 cycles), the histogram plot for the (b) SET (c) RESET, (d) the endurance (V_READ_ = 0.1V) and (e) the cumulative probability distribution curve for the LRS/ HRS data of an arbitrarily chosen device in the crossbar array.

**Fig. S23** I-V characteristic of 12 arbitrarily chosen devices in a 10x10 crossbar array with 10um^2^ device area.

**Fig. S24** The variation across SET and RESET voltages for 10 devices in a crossbar array depicted by (a) the box plot, b) the cumulative probability distribution, and the histogram plot of variation, for the (c) SET and (d) RESET voltages across the multiple devices.

**References**

[1] M. Qi, R. Z. Xu, G. L. Ding, K. Zhou, S. R. Zhu, Y. B. Leng, T. Sun, Y. Zhou, S. T. Han, *Mater Horiz* **2024**, 11, 939.

[2] Z. Y. Lv, S. R. Zhu, Y. Wang, Y. Y. Ren, M. T. Luo, H. N. Wang, G. H. Zhang, Y. B. Zhai, S. L. Zhao, Y. Zhou, M. H. Jiang, Y. B. Leng, S. T. Han, *Adv Mater* **2024**, 36, 2405145.

[3] Y. M. Sun, B. X. Li, M. Liu, Z. K. Zhang, *Mater Today Adv* **2024**, 23, 100515.

[4] L. L. Liu, P. A. Dananjaya, M. Y. Chee, G. J. Lim, C. X. X. Lee, W. S. Lew, *Acs Appl Mater Inter* **2023**, 15, 29287.

[5] V. Raja, K. Hadiyal, A. K. Nath, L. R. Viannie, P. Sonar, J. Molina-Reyes, R. Thamankar, *Materials Science and Engineering: B* **2021**, 264, 114968.

[6] X. H. Zhang, X. N. Zhao, X. Y. Shan, Q. L. Tian, Z. Q. Wang, Y. Lin, H. Y. Xu, Y. C. Liu, *ACS Appl Mater Inter* **2021**, 13, 28555.

[7] G. Milano, F. Raffone, M. Luebben, L. Boarino, G. Cicero, I. Valov, C. Ricciardi, *Acs Appl Mater Inter* **2020**, 12, 48773.

[8] X. Liu, C. Zhang, E. Li, C. Gao, R. Wang, Y. Liu, F. Liu, W. Shi, Y. Yuan, J. Sun, Y.-F. Lin, J. Chu, W. Li, *Advanced Functional Materials* **2024**, 34, 2309642.

[9] Y. Wang, Y. Gong, L. Yang, Z. Xiong, Z. Lv, X. Xing, Y. Zhou, B. Zhang, C. Su, Q. Liao, S.-T. Han, *Advanced Functional Materials* **2021**, 31, 2100144.

[10] T. Guo, J. W. Ge, Y. X. Jiao, Y. C. Teng, B. Sun, W. Huang, H. Asgarimoghaddam, K. P. Musselman, Y. Fang, Y. N. Zhou, Y. A. Wu, *Mater Horiz* **2023**, 10, 1030.
